# Day-ahead active and reactive power scheduling of wind turbines and battery energy storage systems for power loss and CO_2_ emission reduction in AC microgrids

**DOI:** 10.1371/journal.pone.0353697

**Published:** 2026-07-17

**Authors:** Daniel Sanin-Villa, Héctor Pinto Vega, Carlos R. Baier, Walter Gil-González, Luis Fernando Grisales-Noreña

**Affiliations:** 1 Universidad EAFIT, Área de Industria, Materiales y Energía, Medellín, Colombia; 2 Facultad de Ingeniería, Departamento de Ingeniería Eléctrica, Universidad de Talca, Curicó, Chile; 3 Department of Electrical Engineering, Universidad Tecnológica de Pereira, Pereira, Colombia; 4 Grupo de Investigación en Alta Tensión (GRALTA), Escuela de Ingeniería Eléctrica y Electrónica, Facultad de Ingeniería, Universidad del Valle, Cali, Colombia; Asia Pacific University of Technology & Innovation, MALAYSIA

## Abstract

This paper proposes a day-ahead scheduling framework to analyze and optimize the impact of the coordinated active and reactive power management of wind turbines (WTs) and battery energy storage systems (BESSs) on the energy losses and CO_2_ emissions of AC microgrids (MGs). Within this framework, the BESS plays a central role by absorbing surplus renewable generation, mitigating curtailment, supporting voltage regulation, and ensuring a stable and reliable dispatch over a 24-hour horizon. A population-based genetic algorithm (PGA) is proposed as the main solution methodology, while particle swarm optimization (PSO) and the multiverse optimizer (MVO) are employed as benchmark methods for comparison. To ensure a fair assessment, all optimization techniques are implemented under the same parallel processing scheme, using the same decision-variable encoding, feasibility correction procedure, and hourly sequential AC power-flow method. The objective is to minimize network energy losses and CO_2_ emissions under both grid-connected and islanded operating modes. The proposed methodology is validated on 33-node and 69-node MGs, both evaluated under variable demand and wind-generation scenarios to capture the uncertainty and temporal variability associated with renewable production and load behavior. In addition, the BESS model includes charging/discharging efficiency, self-discharge effects, and battery lifetime assessment under the proposed operating scenarios, allowing a more realistic representation of storage performance. The optimization methods are evaluated over 100 independent runs using the best solution, average solution, standard deviation, and computational time as performance indicators. The results show that the proposed PGA provides the most robust and repeatable performance, while also highlighting the operational contribution of the BESS, reducing renewable curtailment, and guaranteeing compliance with all technical constraints, under deterministic baseline operation and under uncertain time-varying operating conditions in both test systems.

## 1 Introduction

The ongoing decarbonization of power systems has spurred the deployment of alternating current (AC) microgrids (MGs) that integrate renewable generation, storage technologies, and advanced scheduling strategies to enhance technical performance and reduce greenhouse gas emissions. In these architectures, wind turbines (WTs) and battery energy storage systems (BESSs) are particularly attractive, as they can be coordinated to provide both active and reactive power support under grid-connected and islanded conditions. Recent reviews emphasize that MG operation is constrained by a complex interaction between network limits, control hierarchies, and environmental targets, and that energy management in this context must address both cost and carbon dioxide (CO_2_) reduction objectives [1]. At the same time, reliability and power quality aspects must be considered from the planning and operation stages, particularly in autonomous or weakly interconnected systems [2].

Several studies have analyzed MGs integrating wind generation and storage to improve power loss performance, voltage profiles, and economic indicators. Early contributions showed that inadequate sizing or siting of storage devices may increase losses and jeopardize voltage stability, whereas optimal coordination of distributed generators and BESS can significantly reduce real and reactive power losses in distribution feeders [3]. More recent works extend these ideas to detailed energy management systems that couple day ahead scheduling, active and reactive power dispatch, and curtailed operation of renewable units. For instance, an energy management system for a low-voltage microgrid MG in Italy, including photovoltaic units, a WT, a BESS, and a combined heat and power plant was proposed with coupled active and reactive power management, showing how inverter capability curves and reactive power penalties influence economic and technical outcomes [4]. In parallel, real-time active-reactive optimal power flow formulations have been developed for distribution networks with wind power and battery storage, where BESS’s flexible operation is used to handle short-term wind variability while respecting multi-phase and multi-timescale constraints [5]. Additionally, MGs have been studied as providers of ancillary services, such as phase balancing and reactive power support, using grid-tied inverters for photovoltaics and storage, with experimental evidence that coordinated reactive control can enhance power quality and reduce losses at the point of common coupling [6].

Optimization-based energy management of BESS in AC MGs has become a central research topic. Recent works have demonstrated that appropriate scheduling of BESS can simultaneously improve technical, economic, and environmental indicators. A cost optimization study in AC MGs with distributed WTs compared population-based genetic algorithm (PGA), particle swarm optimization (PSO), JAYA, and generalized normal distribution optimizer (GNDO), showing that the genetic approach achieved the best performance for operational cost reduction in both grid-connected and isolated configurations [7]. Other contributions have focused specifically on BESS scheduling under multi-objective criteria. An intelligent energy management strategy for BESS based on the GNDO considered the simultaneous minimization of costs, losses, and emissions in on-grid and off-grid scenarios, and reported that this algorithm consistently outperformed continuous genetic algorithms, PSO, and JAYA in terms of solution quality and robustness [8]. A tuned PGA for BESS operation in AC MGs has also been proposed, minimizing operational costs, power losses, and carbon footprint in grid-connected and islanded topologies, and evidencing superior accuracy and stability when compared with PSO and the Vortex Search Algorithm [9]. In a related line, a coordinated active-reactive scheduling framework for BESS in AC MGs was introduced to independently minimize energy losses and CO_2_ emissions, using a PSO implementation and a full AC power-flow model that explicitly accounts for the active and reactive dispatch of storage units [10].

The integration of BESS to reduce losses and emissions in distribution networks has also been addressed by several authors. A master slave methodology based on a parallel discrete Vortex Search Algorithm for siting and sizing of lithium-ion batteries, combined with PSO for operation, was applied to a 33-node grid-connected network with photovoltaic generation, achieving noticeable reductions in energy losses and CO_2_ emissions compared with multiple benchmark approaches [11]. Extending this perspective, a Multiverse Optimizer (MVO) combined with a matrix power flow method was used to optimally integrate photovoltaic distributed generators and D-STATCOMs, targeting annual investment and operating cost minimization and showing that the multiverse strategy presented the best trade-off between solution quality and processing time when compared with several alternative metaheuristics [12]. A related contribution proposed an amended MVO for sizing hybrid MGs with energy storage, demonstrating competitive performance on cost and renewable penetration indicators compared with other advanced algorithms [13]. More recently, an energy management system based on a parallel implementation of the MVO with successive approximations (SA) was applied to BESS scheduling in AC MGs, reporting significant reductions in energy losses and CO_2_ emissions in both grid-dependent and self-powered modes, and superior consistency compared with previously reported methods [14].

Other studies have combined wind generation, BESS, and additional devices such as D-STATCOMs, evaluating economic and technical performance under different metaheuristic strategies. A smart energy strategy for AC MGs that integrates wind generation, BESSs, and D-STATCOMs used a Gray Wolf Optimizer coupled with SA in a master slave structure, with the objective of minimizing operational costs in grid connected and islanded modes and ensuring voltage regulation and current limits, and the results indicated that this approach outperformed PSO and classical genetic algorithms in terms of cost and stability [15]. More general frameworks for economic emission dispatch in MGs have been addressed using improved Mayfly Optimization, Whale Optimization, and other metaheuristics, focusing on combined cost and emission objectives in systems that mix conventional and renewable sources [16,17]. Multi-objective scheduling of MGs integrated into virtual power plants with photovoltaic, wind, storage, and combined cooling, heating, and power units has been studied using enriched PSO variants to reduce operating costs and net emissions simultaneously [18]. In the context of hybrid wind-photovoltaic systems, PSO combined with NSGA-II has been used to design optimal capacities to reduce cost, maximize renewable consumption, and limit load shortages [19]. Similarly, PSO and Electric Eel Foraging Optimization have been applied to grid-connected wind photovoltaic hybrid systems in order to improve power regulation, mitigate loading issues, and enhance grid integration quality [20]. Additional contributions examine multi-objective power scheduling in residential MGs with vehicle-to-grid strategies and demand response, with an emphasis on economic and emission indicators [21].

From a technical perspective, a wide literature addresses power loss reduction and voltage profile enhancement in distribution networks through optimal placement of distributed generators or network reconfiguration. An improved PSO methodology for optimal allocation of multiple DG units in radial networks showed significant reductions in both real and reactive power losses and substantial voltage profile improvements [22]. Hybrid metaheuristic reconfiguration of radial systems, for example, via Levy Flight-based Aquila Optimization, has been proposed to minimize power loss while enhancing the voltage profile in the standard IEEE 33- and 69-bus systems [23]. MG integration and storage management have also been studied from a reliability perspective, where genetic algorithms are used to optimize energy exchanges between MGs and the main grid in order to reduce congestion, penalties, and line overloads, leading to improved reliability coefficients and reduced operating costs for distribution system operators and MG owners [24]. In addition, several studies address reactive power optimization in distribution networks with wind power integration. For example, an improved multi-objective PSO with adaptive grids and external archives has been applied to reactive power optimization in the IEEE 33-bus system with wind generation, targeting the simultaneous minimization of active power losses and voltage deviation, and demonstrating a better Pareto front quality than NSGA-II [25].

To align this study with the principles of advanced energy storage research, special emphasis is placed on the role of the BESS within the MG [26]. Rather than addressing the broader behavior of the distribution network, the analysis centers on the BESS’s performance, operational flexibility, and active–reactive support capabilities, which enable improved utilization of renewable energy and the enforcement of operational constraints. The coordinated interaction between the BESS and the WT is thus examined as the core mechanism for mitigating curtailment, enhancing technical feasibility, and strengthening the overall energy management strategy [27].

The body of research summarized above indicates that AC MGs, particularly those with wind generation and BESS, provide a suitable platform to address both technical performance and environmental goals. Cost-oriented formulations are dominant, with many studies focusing on minimizing operating costs and reporting losses and emissions as secondary indicators [7–9,15]. Emission-related objectives are typically combined with economic criteria in single or composite multi-objective frameworks, as illustrated in combined economic emission dispatch for MGs and stand-alone systems with demand response [16,17]. Only a limited set of contributions has treated energy losses and CO_2_ emissions as independent objective functions with full AC modeling and daily scheduling horizons [10,14]. Moreover, most existing works either focus on storage operations with fixed wind generation profiles or analyze wind-based distributed generators, prioritizing cost minimization rather than studying the joint active and reactive operation of WTs and BESSs for the separate optimization of losses and emissions in both grid-connected and islanded regimes.

In terms of optimization techniques, PSO and its variants remain a reference method for MG operation, dispatch, and planning problems [3,11,18–20,25]. Multiverse-based strategies have recently shown promising results in MG-related applications, both for device integration and for BESS scheduling [12–14]. PGAs, including tuned parallel implementations, have proven effective in AC MGs with WTs and BESSs, particularly in terms of cost and multi-objective performance [7,9]. However, a direct and systematic comparison among a tuned PGA, a parallel MVO, and a parallel PSO in the specific context of WTs and BESSs coordinated active reactive scheduling with two independent objectives, power loss minimization and CO_2_ emission minimization, has not yet been reported. Existing studies usually prioritize cost as the main objective, restrict reactive power support to a subset of devices, or treat grid-connected and islanded modes separately rather than within a unified analytical framework.

The literature also shows that uncertainty treatment remains a relevant aspect in WT-BESS scheduling. Recent studies have analyzed the impact of false data injection on WT performance, showing that wind-based systems can be affected by distorted measurements and data integrity disturbances [28]. Other works have focused on smoothing WT power output in MGs using ultracapacitor-based storage and continuous wind speed forecasting [29], as well as on increasing harvested wind energy through forecasting-assisted control strategies [30]. These contributions reinforce the need to evaluate WT-BESS scheduling frameworks under variable wind and demand conditions instead of relying only on deterministic profiles.

In this context, the present research proposes a day-ahead energy management and scheduling framework for AC MGs that coordinates the explicit active and reactive power dispatch of WTs and BESSs under both grid-connected and islanded operating modes. The proposed framework is designed to analyze and optimize the impact of such coordinated operation on two key network-level performance indicators, namely energy losses and CO_2_ emissions, while preserving technical feasibility over a 24-hour horizon.

The contribution of this work is defined by the joint treatment of several elements within a unified scheduling framework. First, the proposed model explicitly coordinates active and reactive power management for both WTs and BESS units, enabling the study of their combined effects on network losses, emissions, and operational feasibility. Second, it formulates two independent optimization problems, one aimed at minimizing energy losses and the other focused on minimizing CO_2_ emissions, instead of relying on weighted aggregate functions. Third, the same formulation is evaluated under both grid-connected and islanded operating conditions. Fourth, the BESS model incorporates charging/discharging efficiency, self-discharge effects, and a first-order throughput-based lifetime assessment under the proposed operating scenarios. Fifth, the methodology is validated in both 33-node and 69-node AC MGs, and both systems are evaluated under varying wind generation and demand scenarios to capture uncertainty and temporal variability. Finally, a tuned PGA is proposed as the main solution methodology, while PSO and MVO are used as benchmark methods under the same decision-variable encoding, feasibility-correction strategy, parallel-processing structure, hourly sequential AC power-flow evaluation, and 100-run statistical assessment. To the best of the authors’ knowledge, this exact combination has not been previously reported for the coordinated day-ahead active and reactive scheduling of WT-BESS units in AC MGs.

The use of two independent single-objective formulations is intentional. This decoupled structure makes it possible to separately identify the operating pattern most favorable for energy-loss reduction and the one most effective for CO_2_ mitigation, without introducing subjective weighting factors or Pareto-ranking decisions. As a result, the proposed design improves the interpretability of the obtained schedules and enables a cleaner and fairer algorithmic comparison, since all methods solve exactly the same formulation in each operating scenario. Although the two objectives are solved independently in this study, practical operation may require reconciling the schedules obtained for loss minimization and CO_2_ emission reduction. This reconciliation can be performed through Pareto-based decision-making, weighted preference factors defined by the operator, or lexicographic dispatch rules in which one objective is prioritized while the other is constrained within an acceptable range. Therefore, the independent formulation adopted here should be interpreted as a diagnostic and benchmarking stage that identifies the limiting schedules for each objective before a final operational decision is made.

The remainder of the manuscript is structured as follows. First, the AC MG model, including WT and BESS representations and the CO_2_ emission formulation, is presented. Then, the optimization problems for power loss minimization and emission minimization are introduced, together with the proposed metaheuristic solution schemes. Next, the test system, simulation scenarios, and performance metrics used to compare PGA, MVO, and PSO are described. Subsequently, numerical results are reported and discussed for grid-connected and islanded operation, highlighting the trade-offs between losses, emissions, and algorithmic behavior. Then, the effectiveness of the proposed strategy is evaluated on a more complex test system, considering both grid-connected and islanded operating modes. Finally, conclusions and possible extensions are outlined.

## 2 Mathematical model

The optimization variables of the metaheuristic layer are the hourly active and reactive power setpoints of the WTs and BESS units, namely $P_{w,i,t}$, $Q_{w,i,t}$, $P_{B,j,t}$, and $Q_{B,j,t}$. In contrast, bus-voltage magnitudes, voltage angles, slack-bus power, line currents, and the SOC trajectory are dependent state variables obtained after the feasibility correction and the sequential AC power-flow evaluation of each candidate schedule. All evaluations are performed over a 24-hour horizon, consistent with the MATLAB implementation of hourly sequential power flows.

The MG is modeled as a balanced radial distribution system represented by its admittance matrix $Y_{bus}$. Let $V_{n,t}$ and $\theta_{n,t}$ denote the voltage magnitude and angle at bus *n*. The active and reactive power balances at every bus are enforced through the AC power flow equations


$$\begin{array}{c} P_{n,t}=\sum_{m\in 𝒩}V_{n,t}V_{m,t}\left(G_{nm}\cos\theta_{nm,t}+B_{nm}\sin\theta_{nm,t}\right), \end{array}$$ (1)

$$\begin{array}{c} Q_{n,t}=\sum_{m\in 𝒩}V_{n,t}V_{m,t}\left(G_{nm}\sin\theta_{nm,t}-B_{nm}\cos\theta_{nm,t}\right), \end{array}$$ (2)

where $G_{nm}$ and $B_{nm}$ are the conductance and susceptance entries of $Y_{bus}$, and $\theta_{nm,t}=\theta_{n,t}-\theta_{m,t}$. For buses equipped with WT or BESS units, the net injected power equals the sum of the devices’ contributions minus the local demand.

WTs follow their corresponding power curve, so the active generation satisfies


$$\begin{array}{c} 0\leq P_{w,i,t}\leq P_{w,i,t}^{\max}, \end{array}$$ (3)

where $P_{w,i,t}^{\max}$ is calculated from the wind-speed profile. Reactive power capability is modeled through the converter’s apparent power limit,


$$\begin{array}{c} |Q_{w,i,t}|\leq \sqrt{(S_{w,i}^{\max}{)}^{2}-P_{w,i,t}^{2}}. \end{array}$$ (4)

The BESS injections incorporate charge and discharge limits obtained from the nominal battery energy and the maximum charging and discharging durations used in the MATLAB code, yielding


$$\begin{array}{c} P_{B,j}^{\min}\leq P_{B,j,t}\leq P_{B,j}^{\max}. \end{array}$$ (5)

The inverter capability imposes an additional bound:


$$\begin{array}{c} |Q_{B,j,t}|\leq \sqrt{(S_{B,j}^{\max}{)}^{2}-P_{B,j,t}^{2}}. \end{array}$$ (6)

The state of charge evolves according to


$$\begin{array}{c} {SOC}_{j,t+1}={SOC}_{j,t}-\phi_{j}P_{B,j,t}, \end{array}$$ (7)

where $\phi_{j}$ is a charge-discharge coefficient dependent on battery size and time constants. Operational feasibility requires


$$\begin{array}{c} {SOC}_{j}^{\min}\leq {SOC}_{j,t}\leq {SOC}_{j}^{\max},\;{SOC}_{j,24}={SOC}_{j,0}, \end{array}$$ (8)

ensuring that the daily schedule is cyclic.

Network security is enforced through voltage limits at every bus,


$$\begin{array}{c} V_{\min}\leq V_{n,t}\leq V_{\max}, \end{array}$$ (9)

and through ampacity constraints on every line, implemented through the line currents obtained from the hourly power flow. Any violation is penalized within the objective function, consistent with the MATLAB penalty scheme applied to voltage deviations, line overloads, and apparent power violations.

A key element of the formulation is the modeling of the slack bus in the two operating modes.

In grid-connected mode, the slack bus may either import or export active power. Therefore, two emission indicators are distinguished. The imported grid emissions are computed as


$$\begin{array}{c} E_{CO_{2},t}^{imp}=\alpha_{grid}{\left[P_{slack,t}\right]}_{+}, \end{array}$$ (10)

where ${\left[x\right]}_{+}=\max(0,x)$. In addition, the net emission balance is defined as


$$\begin{array}{c} E_{CO_{2},t}^{net}=\alpha_{grid}P_{slack,t}. \end{array}$$ (11)

Under this convention, positive values indicate emissions associated with imported energy, whereas negative values indicate an export credit associated with renewable surplus delivered to the upstream grid. Therefore, negative values do not represent negative physical emissions, but rather a net accounting balance under export conditions.

In islanded mode, the slack bus represents a local diesel generator constrained to operate within the admissible loading interval reported in the test scenario. Therefore,


$$\begin{array}{c} 0.40\;P_{\text{slack}}^{rated}\leq P_{\text{slack},t}\leq 0.80\;P_{\text{slack}}^{rated},\;\forall t\in 𝒯. \end{array}$$ (12)

Since no external power can be imported, feasibility depends entirely on the coordinated operation of the WTs, the BESS fleet, and the dispatchable unit.

To avoid ambiguity, the hourly active power loss is obtained from the branch power flows computed by the AC power-flow solution:


$$\begin{array}{c} P_{t}^{loss}=\sum_{\ell \in ℒ}\left(P_{\ell ,t}^{from}+P_{\ell ,t}^{to}\right), \end{array}$$ (13)

where $P_{\ell ,t}^{from}$ and $P_{\ell ,t}^{to}$ denote the active powers at the sending and receiving ends of branch ℓ, respectively. Under the adopted sign convention, this quantity is equivalent to the system-wide generation-demand mismatch after the AC power-flow solution.

Two independent optimization problems are solved for each operating mode. The first minimizes the daily active power losses,


$$\begin{array}{c} \min\;P_{\text{loss}}^{day}, \end{array}$$

while the second minimizes the total daily CO_2_ emissions,


$$\begin{array}{c} \min\;E_{CO_{2}}^{day,mode}. \end{array}$$

Both problems use the same set of constraints and differ only in their objective functions and slack-bus interpretation. The complete evaluation of each candidate solution is performed through an hourly AC power flow, and feasibility is enforced using the penalty structure implemented in the MATLAB routines, as presented in the Equation 14:


$$\begin{array}{c} F_{f}=O_{f}+Penalty. \end{array}$$ (14)

In this Equation, $F_{f}$ corresponds to the fitness function, which is a penalized representation of the objective function to be minimized. $O_{f}$ denotes the objective function to be optimized, either associated with power losses or emissions, while *Penalty* represents the penalty term applied when any system constraint is violated. These constraints are assigned a fixed penalty value of 1000 per violation and may include loading limits, voltage limits, and/or violations of the diesel generator output limits in the off-grid operation mode of the MG. The penalty coefficient was kept fixed for all algorithms, operating modes, and objective functions to preserve a homogeneous comparison. The value of 1000 does not represent a physical cost; rather, it acts as a feasibility-enforcement factor that makes infeasible schedules less competitive than feasible alternatives. The fixed penalty was used to ensure that all methods were evaluated under the same constraint-handling rule.

## 3 Methodology

The proposed methodology integrates a detailed operational model of the MG with a population-based optimization framework. The central idea is to evaluate hourly active and reactive power schedules for the WTs and BESS units over a full 24-hour horizon. Each candidate solution contains the complete set of decision variables that define the operation of all distributed resources throughout the day.

The first step is the codification of the solution vector. Each candidate is represented as a one-dimensional array that stores all hourly setpoints for the distributed resources. The WTs contribute 24 active-power values and 24 reactive-power values per unit. The BESS units contribute 24 active-power values and 24 reactive-power values per unit. The complete schedule is represented as:


$$\begin{array}{c} 𝐱=\{P_{w,1,t},\ldots ,P_{w,3,t},\;\ldots ,\;Q_{w,1,t},\ldots ,Q_{w,3,t},\\ \ldots ,\;P_{B,1,t},\ldots ,P_{B,3,t},\;\ldots ,\;Q_{B,1,t},\ldots ,Q_{B,3,t}{\}}_{t=1}^{24}. \end{array}$$ (15)

This matches the structure used in the MATLAB implementation. This representation contains 288 variables and defines the entire operational profile to be evaluated by the optimization method.

Once a candidate vector is generated, a feasibility mechanism adjusts the batteries’ active power schedules to satisfy the charge and discharge limits and maintain the state of charge within the admissible range. This adjustment procedure ensures consistency with the recursive SOC equation and a valid transition between consecutive hours. Candidates that violate these requirements are corrected before the power flow evaluation.

After feasibility verification, the 24-hour assessment is performed using sequential AC power-flow calculations. At each hour, the nodal injections derived from the decision vector are applied to the network, and the Newton-Raphson method computes the voltage magnitudes, angles, line currents, and the slack-bus power. Daily active power losses and daily CO_2_ emissions are obtained from the aggregation of hourly results. Voltage violations, current overloads, and converter capacity violations are penalized in the objective function to guide the optimizer toward feasible solutions.

The search process is conducted using three parallel metaheuristic techniques. The PGA serves as a baseline reference because it can preserve structural diversity and effectively explore nonconvex regions. The PGA operates directly on the encoded decision vectors, generating new candidates through selection, recombination, and mutation. Each new candidate is then evaluated using the feasibility routine and the AC power-flow procedure. The process repeats until the population converges or the maximum number of generations is reached.

For the three metaheuristics, the dominant computational cost is the repeated evaluation of candidate schedules through 24 sequential AC power-flow solutions. If $N_{p}$ denotes the population size, $N_{i}$ the number of iterations, and $C_{PF}$ the cost of one hourly AC power-flow solution, then the computational effort can be expressed in the order of $𝒪(N_{p}N_{i}\times 24\times C_{PF})$. Differences among PGA, PSO, and MVO arise mainly from their update operators, whereas the fitness-evaluation stage dominates the execution time. In addition, candidate evaluations are mutually independent within each iteration, which makes the framework naturally suitable for parallel implementation. The computational times reported in the Results and Discussion section correspond to the total wall clock time required to complete each optimization run. Therefore, each reported value includes candidate decoding, SOC feasibility correction, the 24 sequential hourly AC power flow evaluations, penalty calculation, objective function aggregation, and the algorithm specific update operations of PGA, PSO, or MVO.

**Fig 1:**
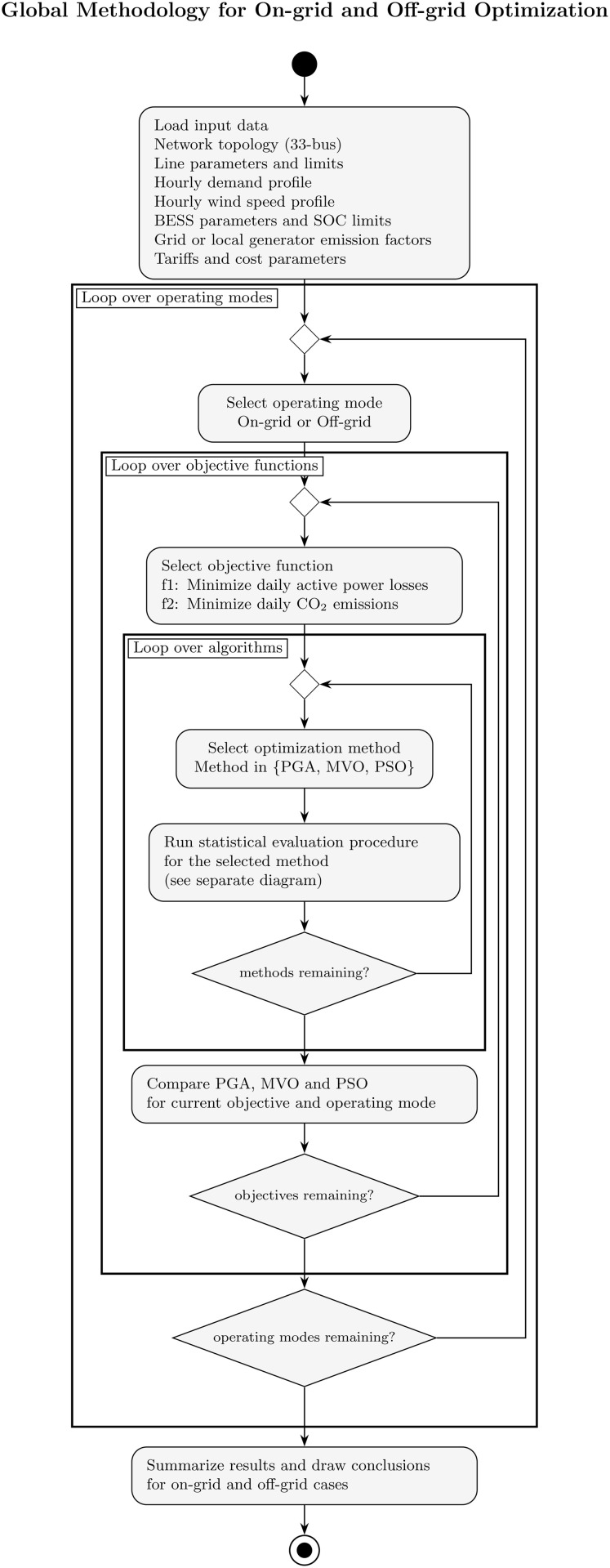
Global methodological framework used in this study. The diagram summarizes the evaluation process for both operating modes, namely the on-grid and off-grid configurations, and for the two independent objective functions associated with power losses and carbon emissions.

Fig 1 presents the global methodological structure adopted in this study. It summarizes the workflow used to assess both operating conditions, namely the on-grid and off-grid configurations, the two independent objective functions related to power losses and carbon emissions, and the statistical evaluation of each algorithm over one hundred independent runs.

### 3.1 Metaheuristic optimization framework

The assessment of scheduling strategies for the MG is conducted using three independent metaheuristic techniques. Each method follows a population-based search scheme in which a set of candidate solutions encodes the hourly active and reactive power injections from WTs and battery storage units. The vector representation is identical across all methods, ensuring that performance differences arise from the algorithms’ intrinsic search dynamics.

The statistical evaluation of each metaheuristic follows a uniform procedure to ensure comparable performance across methods. Each algorithm is executed one hundred independent times under identical operating conditions. For each run, the algorithm initializes its search agents, decodes the corresponding decision vector into hourly active and reactive injections, and applies the feasibility routine to enforce the battery state-of-charge limits. The metaheuristic then iteratively generates, or updates candidate schedules, and each candidate is evaluated through a complete 24-hour AC power-flow simulation. For each candidate schedule, the AC power-flow problem is solved using the Newton–Raphson method. The Newton–Raphson process is stopped when the convergence error falls below 10^−10^ or when a maximum of 1000 internal power-flow iterations is reached. This stopping rule applies only to the AC power-flow solver. The stopping conditions of PGA, PSO, and MVO are instead defined by their tuned population sizes and iteration numbers reported in Table 1.

**Table 1:** Tuned parameters for the optimization methodologies implemented in the 33-node MG (on-grid and off-grid modes).

Methodology	Parameter	Grid-Connected	Isolated
PGA	No. of individuals	53	45
	No. of iterations	10000	10000
	No. of mutations	1	1
PSO	No. of individuals	249	220
	No. of iterations	1800	1957
	Minimum inertia	0.5183	0.4531
	Maximum inertia	1	1
	Cognitive factor	2	2
	Social factor	1.2767	1.0345
	Velocity limit factor	0.0407	0.0643
MVO	No. of individuals	80	69
	No. of iterations	1700	1921
	Min. WEP	0	0
	Max. WEP	0.0303	0.0523
	*p*	20	20

**Fig 2:**
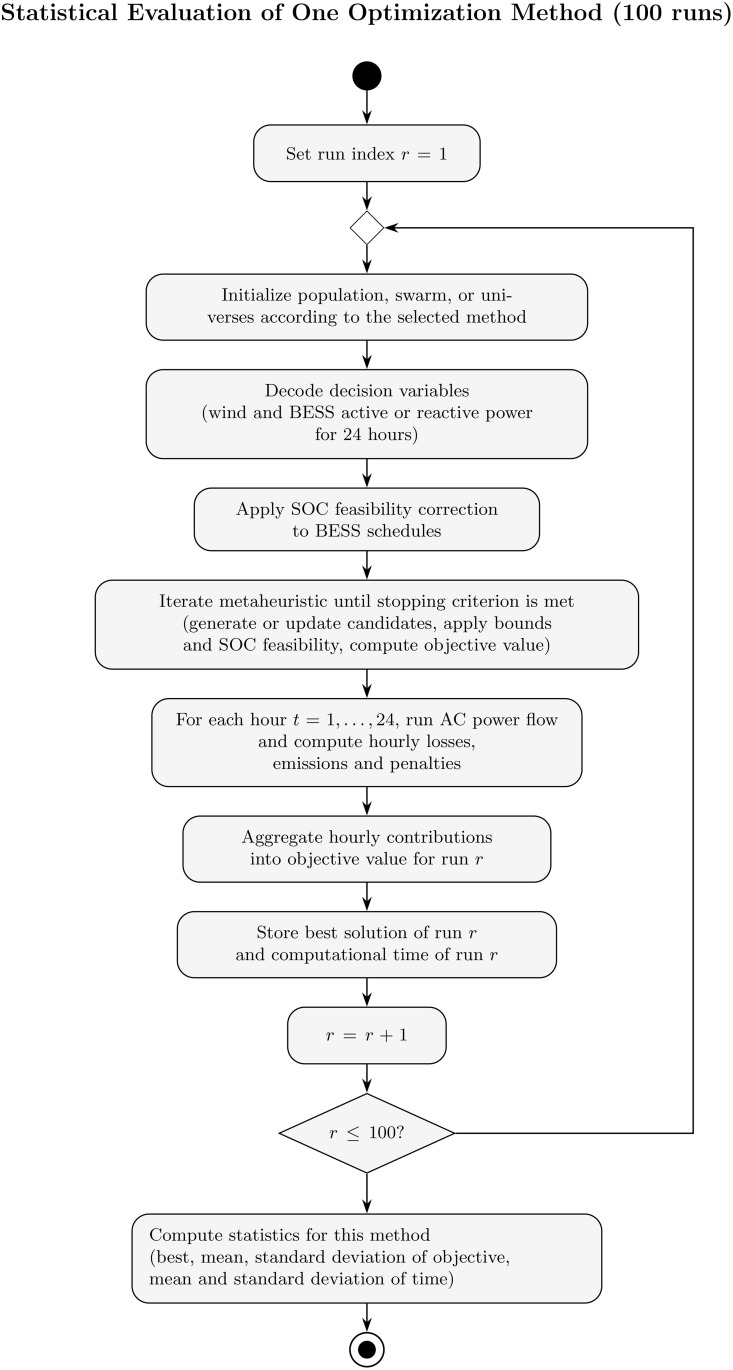
Statistical evaluation process for each optimization method.

The best solution and the corresponding computational time are stored for each run. After completing the 100 runs, the method’s performance is characterized by four indicators: the best objective value obtained, the mean objective value, the standard deviation across runs, and the mean and standard deviation of computational times. Fig 2 illustrates this evaluation process.

The PGA employed in this work uses tournament selection, arithmetic recombination, and random mutations to explore the decision space. The method balances exploration and exploitation through stochastic operators that modify the genes associated with active and reactive injections. The PSO method models each candidate solution as a particle whose velocity is updated using its own historical record and the swarm’s global best solution. This mechanism creates gradual convergence patterns with fewer random perturbations than evolutionary schemes. The MVO follows a cosmological interpretation in which universes with better inflation rates attract others through white hole and black hole mechanisms, while wormhole perturbations refine the vicinity of the current best universe. These three methods are evaluated using the same hourly AC power-flow routines, the same feasibility corrections for battery state of charge, and the same constraint-handling criteria.

The algorithms are summarized below in structured pseudocode. All of them use the same solution encoding and call the same evaluation routine, which performs 24 sequential AC power-flow computations for each candidate solution.

**Algorithm 1. Candidate Schedule Evaluation**



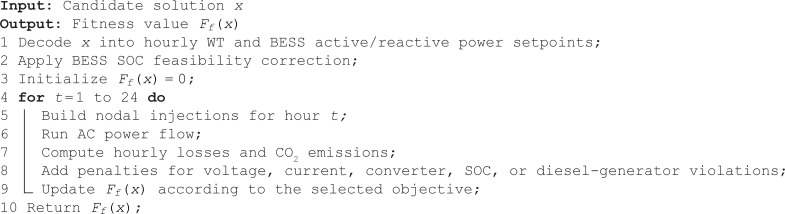



**Algorithm 2. Population-based Genetic Algorithm for Microgrid Scheduling**



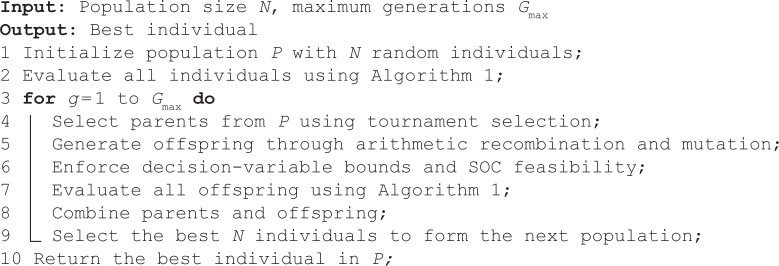



**Algorithm 3. Particle Swarm Optimization for Microgrid Scheduling**



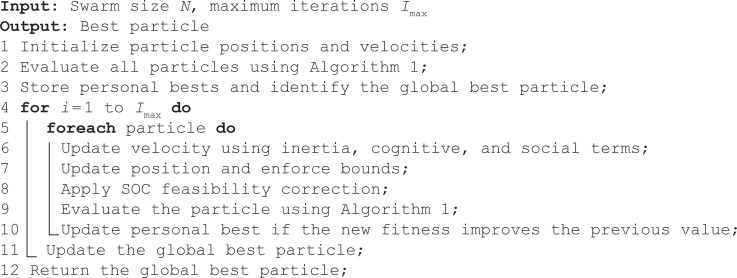



**Algorithm 4. Multi-Verse Optimizer for Microgrid Scheduling**



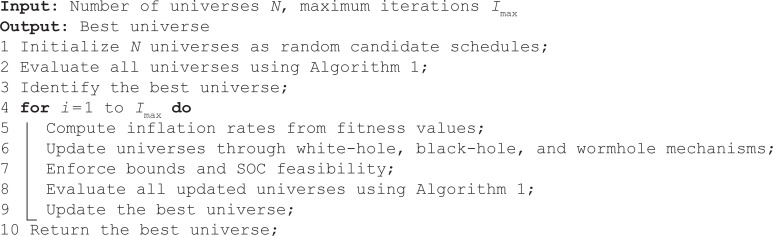



It is important to clarify why deterministic and learning based alternatives were not included as benchmark methods in the present implementation. Deterministic optimization techniques, such as mixed-integer programming, nonlinear programming, or decomposition based approaches, would require additional reformulations of the original AC scheduling problem. In particular, the nonlinear AC power-flow equations, converter capability limits, SOC recursion, grid-connected and islanded slack-bus constraints, and penalty based feasibility handling would generally need convexification, linearization, relaxation, or problem decomposition. These transformations may improve tractability, but they can also alter the feasible region or introduce approximations that make the comparison with population based metaheuristics less direct. For this reason, the present study compares PGA, PSO, and MVO under the same nonlinear AC power-flow evaluation, decision-variable encoding, feasibility correction procedure, and constraint-handling structure.

Learning based optimization or control methods, including reinforcement learning, surrogate assisted optimization, and data driven dispatch policies, also constitute promising alternatives for WT-BESS scheduling. However, their application would require a representative training database, validation under unseen operating conditions, hyperparameter selection, generalization tests, and robustness assessment under scenarios different from those used during training. These requirements would introduce an additional methodological layer beyond the scope of the present benchmarking study, whose objective is to evaluate three population based optimization strategies using identical simulation conditions and repeated independent runs. Therefore, deterministic and learning based methods are identified as relevant extensions for future research, particularly for real-time operation, uncertainty aware dispatch, and implementation in larger distribution feeders.

### 3.2 Parameter specifications for the metaheuristic techniques utilized

To ensure that all employed techniques operate under comparable conditions and achieve optimal performance, their respective parameters were tuned using a PSO algorithm. This PSO configuration consists of a population of 8 particles and a maximum of 300 iterations, with cognitive and social coefficients set to 1.4940, and minimum and maximum inertia weights of 0.0010 and 0.7000, respectively.

The resulting parameter settings for each technique are summarized in Table 1.

### 3.3 Test scenario

#### 3.3.1 33-bus AC microgrid.

**Fig 3:**
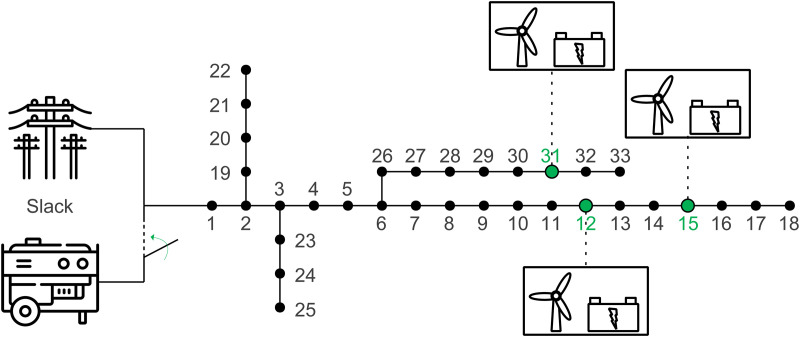
Electrical diagram of the 33-node AC MG.

The proposed optimization framework is evaluated on a distribution-level AC MG derived from the IEEE 33-bus system. The network includes line impedances, thermal limits, transformer connections, and nodal voltage constraints following standard operational requirements for medium-voltage distribution systems, and its technical parameters are obtained from [7]. Two operating modes are considered. In the on-grid configuration, the MG exchanges active and reactive power with the upstream utility grid, subject to a power-factor constraint and real-time tariff signals. In the off-grid configuration, the MG operates autonomously. Instead of the main electrical grid being connected to the slack bus, a diesel generator is used. This generator can inject power into the MG but cannot absorb it, as it lacks parallel energy storage. Furthermore, the diesel generator must maintain its power injection within 40% to 80% of its rated capacity (in this case, 4000 kW) at all times to ensure proper operation and to extend its service life [31]. In this mode, the MG must be capable of self-sustained operation, relying on this generator, along with local generation units and BESS, to maintain system security and stability. A graphical representation of the MG is shown in Fig 3.

The WT model is based on a steady-state aerodynamic power curve with a maximum nominal capacity of 1300 kW. The machine is interfaced with the MG via a power electronic converter that can independently dispatch active and reactive power. The storage device consists of a 2000 kWh lithium-ion battery bank with power limits of ±500 kW and a state-of-charge (SOC) operating band restricted to [10,90] % to avoid accelerated degradation. Pairs of WTs and BESS units can be found at nodes 12, 15, and 31 of the MG, each with the previously described technical specifications.

Time-varying demand is represented by a 24-hour load curve derived from empirical measurements in Colombian distribution networks. In on-grid mode, carbon-intensity coefficients are assigned to imported energy based on regional hourly emission factors. All simulations are performed at one-hour resolution, and AC power-flow computations use the Newton-Raphson method, ensuring exact enforcement of voltage and line-flow limits during the optimization process.

**Fig 4:**
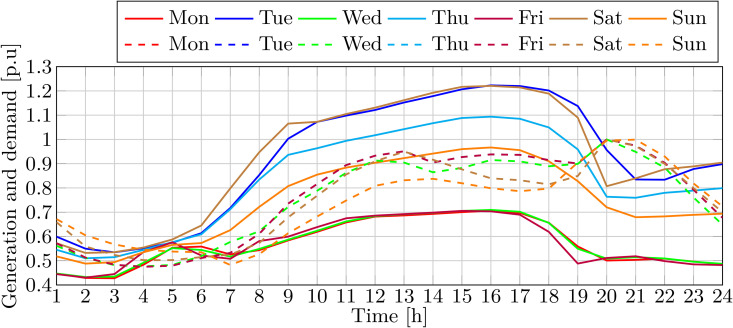
Hourly power demand and generation in the 33-node AC MG.

To address the variability of renewable generation and demand in the 33-node AC MG, an uncertainty analysis was incorporated using differentiated daily profiles of wind generation and load demand, as shown in Fig 4, where solid lines represent the wind generation profiles for each day of the week, while dashed lines correspond to the demand profiles. Each daily scenario preserves the 24-hour scheduling horizon, but modifies the hourly availability of wind power and the nodal demand profile. Thus, the optimization problem is solved independently for each uncertainty scenario while maintaining the same network topology, WT-BESS locations, technical constraints, and algorithmic parameters.

#### 3.3.2 69-bus AC microgrid.

**Fig 5:**
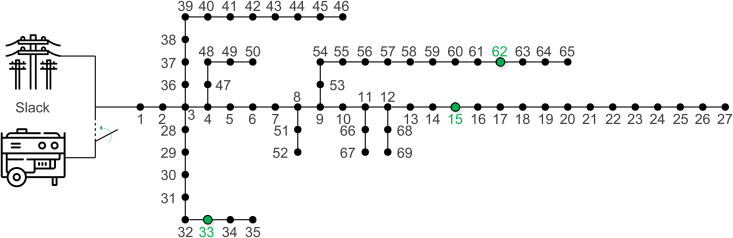
Electrical diagram of the 69-node AC MG.

The second test MG used in this work corresponds to a 69-node AC MG, whose technical parameters are obtained from [32]. Similar to the 33-node AC MG, it can operate in both on-grid and off-grid modes, as shown in Fig 5. For this case, the optimization is performed independently for each day of the week using day-specific 24-hour demand and wind-generation profiles. Therefore, the decision vector structure remains unchanged, while the operating conditions vary from one day to another.

In both operating modes, WT-BESS units are allocated at buses 15, 33, and 62. Each WT has a rated power of 1300 kW, whereas each BESS unit has an energy capacity of 1000 kWh, active power limits of ±250 kW, and SOC bounds between 10% and 90%. Battery lifetime is estimated assuming 2000 life cycles. Additionally, in off-grid operation, a diesel generator is placed at the slack bus following the same criterion adopted for the 33-node MG. In this case, the generator is rated at 2000 kW.

**Fig 6:**
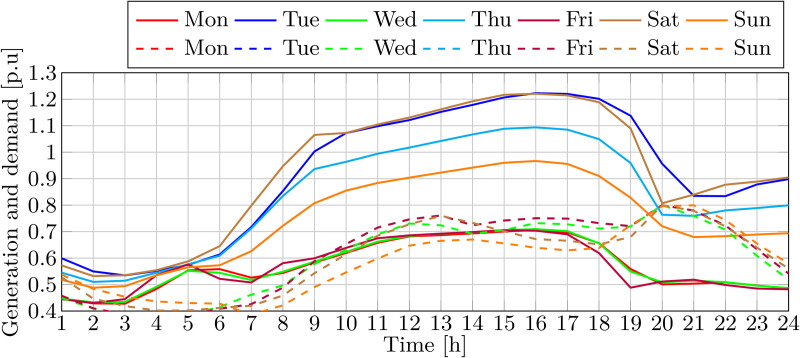
Hourly power demand and generation in the 69-node AC MG.

For this MG, the operation is driven by wind generation and demand profiles from a region in Colombia [33], obtained using artificial neural networks and data from the local distribution company, respectively. These data are presented in Fig 6, where the solid and dashed lines retain the same meaning and graphical convention established in Fig 4. By considering differentiated daily profiles over the week, the analysis incorporates uncertainty in the temporal variability of both demand and renewable generation, thereby providing a more realistic assessment of microgrid operation.

### 3.4 Parameters related to CO_2_ emissions in the MG

To accurately evaluate the impact of each power generation component in the MGs, it is essential to determine its corresponding parameters, particularly those related to CO_2_ emissions. These parameters are summarized in Table 2. In this study, the same emission parameters are used for both the 33-node and 69-node MGs.

**Table 2:** Emissions parameters for the grid-connected and isolated operating modes of the 33- and 69-node MGs.

Parameter	Operation Value	Parameter	Operation Value
$\alpha_{grid}$	0.1644 [kgCO_2_/kWh]	$\alpha_{isl}$	0.2671 [kgCO_2_/kWh]

## 4 Results and discussion

### 4.1 33-bus AC microgrid

The performance of the three metaheuristic methods is examined under two operating conditions in 33-bus test feeder, namely the on-grid and off-grid configurations. For the on-grid case, results are compared with the base case, which corresponds to the original power flow of the 33-node system without optimization. In contrast, the off-grid case does not include a base scenario because the MG depends entirely on local generation and storage. Consequently, the comparison in the islanded mode focuses strictly on the relative performance among the three algorithms.

Table 3 summarizes the key indicators obtained from one hundred independent runs of each method. These include the best solution, the standard deviation expressed as a percentage, the average solution, and the average computational time.

**Table 3:** Energy losses and CO_2_ emissions for on-grid and off-grid operating modes. Results correspond to one hundred independent runs of each algorithm.

Qty	Scen	Metric	PSO	PGA	MVO	Base
Energy losses	On	Min (kWh)	495.21	494.34	495.87	3378.92
		STD (%)	0.48	0.06	0.98	–
		Avg (kWh)	498.23	494.72	500.78	–
		Time (s)	286.28	315.10	167.63	–
	Off	Min (kWh)	545.68	541.46	616.96	–
		STD (%)	1.04	0.47	3.20	–
		Avg (kWh)	553.86	547.15	678.40	–
		Time (s)	293.86	318.98	128.05	–
CO_2_ emissions	On	Min (kg)	4256.07	4139.69	3916.90	12541.20
		STD (%)	2.46	0.47	3.53	–
		Avg (kg)	4551.53	4183.01	4292.82	–
		Time (s)	303.51	321.49	201.05	–
	Off	Min (kg)	10261.97	10257.77	10261.54	–
		STD (%)	1.38	0.05	1.21	–
		Avg (kg)	10457.28	10259.78	10484.42	–
		Time (s)	305.04	326.28	161.88	–

The results show a substantial improvement in both energy losses and emissions for the on-grid configuration. The base case for the 33-node system shows daily losses of 3,378.92 kWh. All algorithms produce reductions exceeding 85 percent, with the PGA achieving the best performance at 494.34 kWh. The PSO and MVO achieve similar values, although the MVO exhibits noticeably higher variability, as indicated by a standard deviation of 0.98 percent. The average daily losses mirror those of the best solutions, with the PGA again yielding the lowest mean value among the three methods.

In the off-grid configuration, there is no reference base case because the system depends entirely on local generation. The comparison is therefore restricted to the three algorithms. In this mode, the PGA provides the best solution, achieving the lowest daily losses at 541.46 kWh with the smallest variability. The PSO also performs consistently, whereas the MVO shows the highest loss values and the largest variability. These patterns suggest that the stability offered by the evolutionary structure of the PGA becomes advantageous under the tighter operational constraints of islanded MGs.

For emissions, the on-grid base case results in 12541.20 kgCO_2_ per day. All algorithms substantially reduce this value. The MVO attains the lowest emission level among the three methods at 3916.90 kgCO_2_, although it exhibits greater dispersion across the 100 runs. The PGA again shows both a low emission level and the smallest variability, indicating a more consistent performance. The PSO converges to slightly higher values but still maintains a significant reduction relative to the base case.

In the off-grid condition, all methods operate under the same structural constraints, and no base comparison is available. The PGA once more provides the most stable behavior, with both the best and average emission levels slightly outperforming those of the PSO and MVO. The higher variability observed in the MVO is consistent with its exploratory dynamics, which tend to generate a wider distribution of solutions in highly constrained operating regimes.

To complement the absolute values reported previously, the percentage reductions in energy losses and CO_2_ emissions relative to the on-grid base case are presented below. These indicators allow a clearer interpretation of the improvement achieved by each algorithm relative to the original operation of the 33-node system.

**Fig 7:**
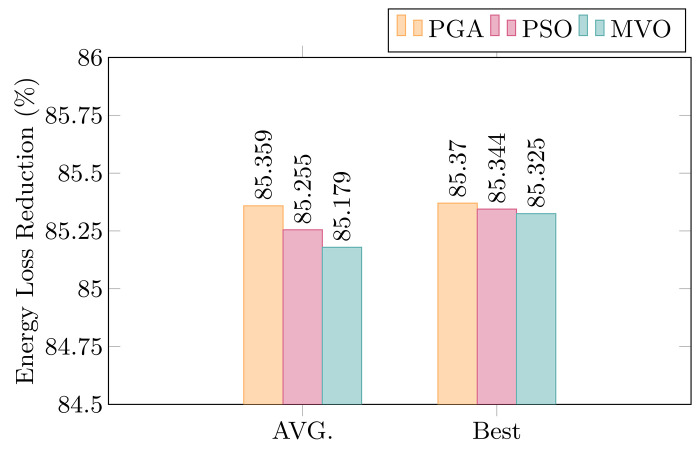
Energy loss reduction in the 33-node system under grid-connected operation. The bars show the best and average percentage reductions with respect to the base case for each algorithm.

For energy losses (Fig 7), the three algorithms achieve reductions above 85%. At the level of best solutions, the PGA reaches the highest reduction at 85.37%, followed very closely by the PSO and the MVO, which obtain 85.34% and 85.32%, respectively. The average values maintain the same ordering, with the PGA again achieving the most favorable performance at 85.36%. These reductions confirm the high effectiveness of the three optimization strategies in mitigating resistive losses under on-grid operation.

**Fig 8:**
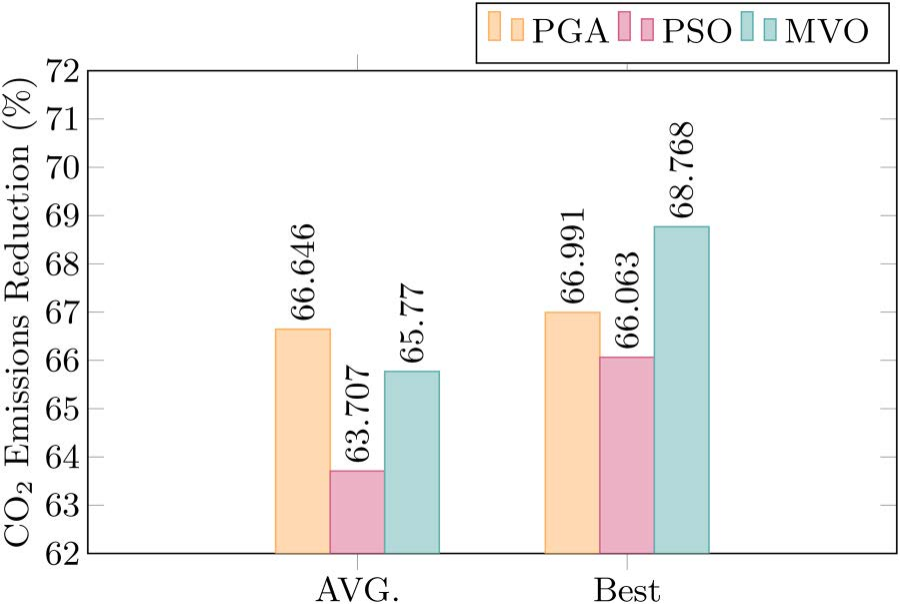
CO_2_ emissions reduction in the 33-node system under grid-connected operation. The bars show the best and average percentage reductions with respect to the base case for each algorithm.

For emissions (Fig 8), the improvements are also significant. The MVO achieves the highest reduction among the best values, at 68.77%, followed by the PGA at 66.99% and the PSO at 66.06%. The average emission reductions show slightly different behavior, with the PGA providing the most stable and favorable result at 66.65%, followed by the MVO at 65.77% and the PSO at 63.71%. These results reveal that the MVO tends to deliver the most aggressive improvements in isolated runs, whereas the PGA exhibits the most consistent behavior across repeated executions.

**Fig 9:**
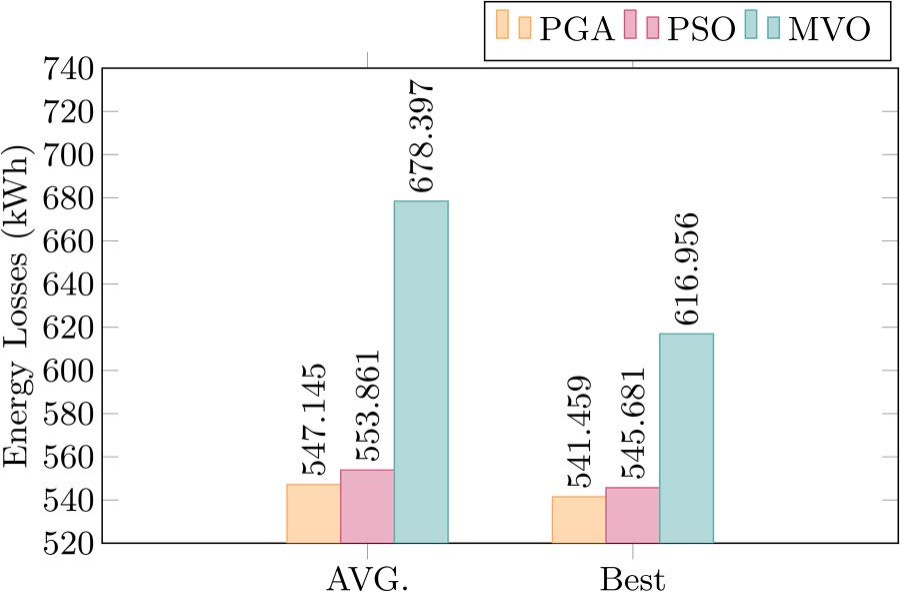
Daily energy losses for the 33-node MG under off-grid operation. The bars show the best and average values obtained over 100 runs of each algorithm. The PGA is used as the reference method, showing the lowest losses and the most favorable average performance compared to PSO and MVO.

**Fig 10:**
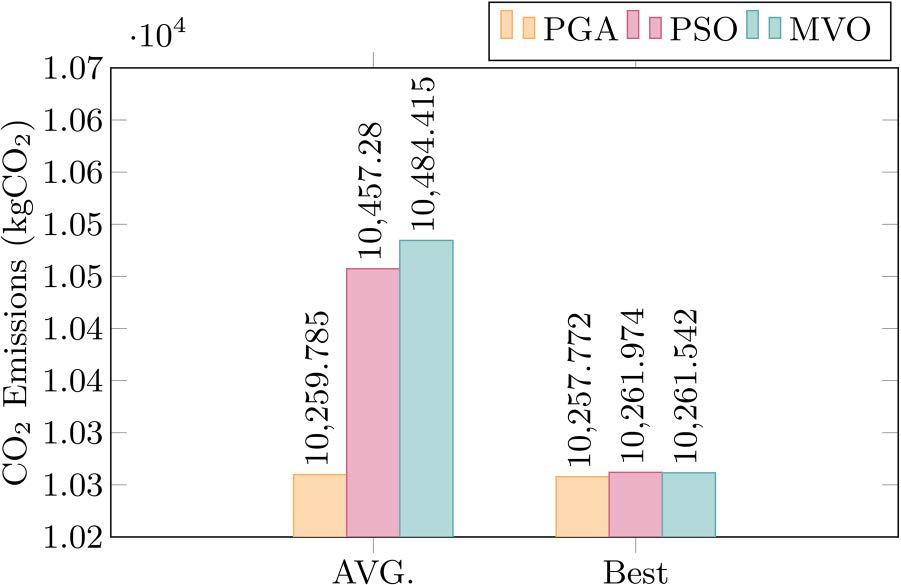
Daily CO_2_ emissions for the 33-node MG under off-grid operation. The bars show the best and average values across one hundred runs. The PGA is again used as a reference and achieves both the lowest emission levels and the smallest gap between best and average performance, while PSO and MVO exhibit higher average emissions.

In the off-grid configuration, the comparison focuses exclusively on the absolute indicators obtained for each algorithm, with the PGA used as the reference method (Fig 9). The following figures present the best and average daily energy losses and CO_2_ emissions for the off-grid 33-node MG, highlighting the relative advantage of the PGA over the PSO and MVO (Fig 10).

**Fig 11:**
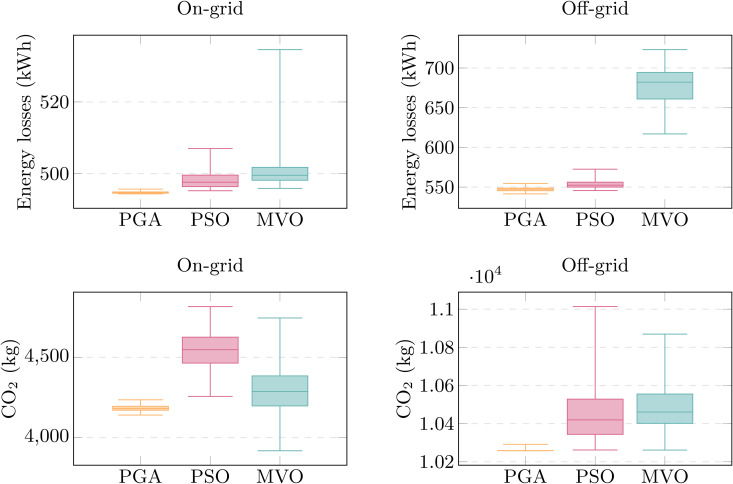
Comparison of energy losses and CO_2_ emissions for on-grid and off-grid modes across the three metaheuristic algorithms over 100 runs.

The comparison among the three algorithms under off-grid operation shows a consistent advantage for the PGA in both performance dimensions. For energy losses, the PGA achieves the lowest daily values and maintains the smallest dispersion between best and average outcomes, indicating a stable and reliable search behavior. The PSO attains similar best values but exhibits greater variability, whereas the MVO yields noticeably higher losses on both indicators. A similar pattern occurs for CO_2_ emissions. The PGA again provides the most favorable and consistent results, whereas the MVO is competitive only in its best run, achieving an emission level comparable to the PSO. However, the MVO’s average emissions remain significantly higher, indicating irregular convergence. These results confirm that the PGA offers a more robust and dependable performance in the off-grid configuration, outperforming the other two methods in both criteria (Fig 11).

This work examines the operational behavior of the MG using the best-performing dispatch obtained from 100 independent optimization runs. For each objective function, the solution with the minimum energy losses and minimum CO_2_ emissions is extracted and analyzed with respect to nodal voltages, branch currents, power exchanges, wind-generation utilization, and BESS dynamics. To facilitate the interpretation of the following figures, it is important to note that the results plotted with dashed lines correspond to the objective function that minimizes CO_2_ emissions, whereas the solid lines represent the objective function that minimizes energy losses.

#### 4.1.1 Voltage profiles, line current flows, and slack bus dispatch.

**Fig 12:**
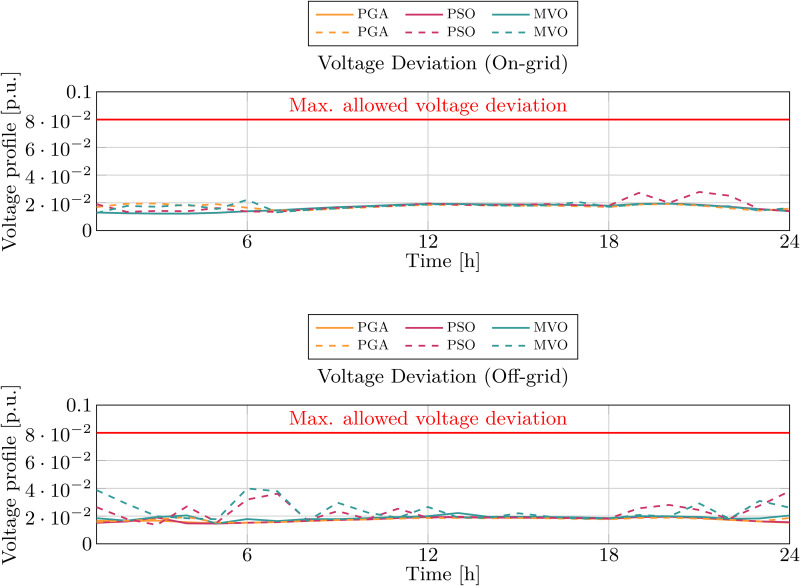
Performance metrics in the 33-node AC MG: voltage deviations for both on-grid and off-grid modes (solid lines correspond to loss minimization, while dashed lines correspond to emission minimization).

**Fig 13:**
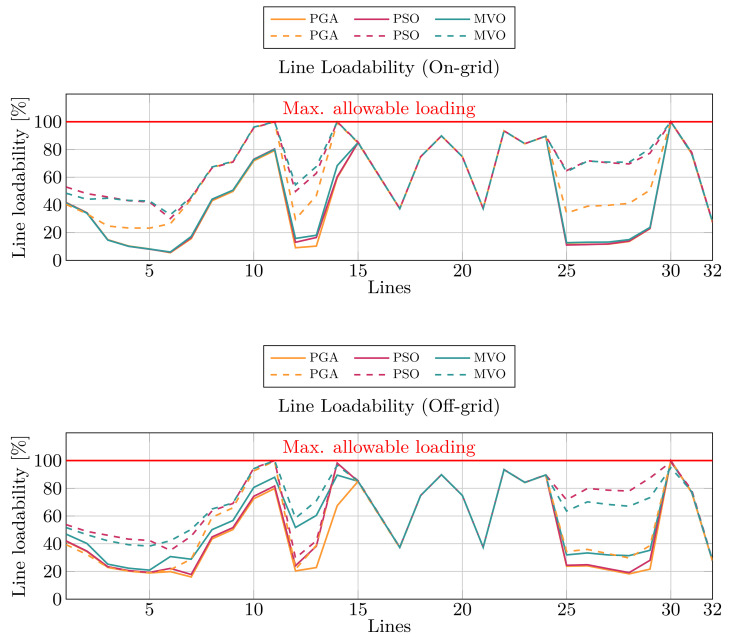
Performance metrics in the 33-node AC MG: Line loadability for both on-grid and off-grid modes, (solid lines correspond to loss minimization, while dashed lines correspond to emission minimization).

**Fig 14:**
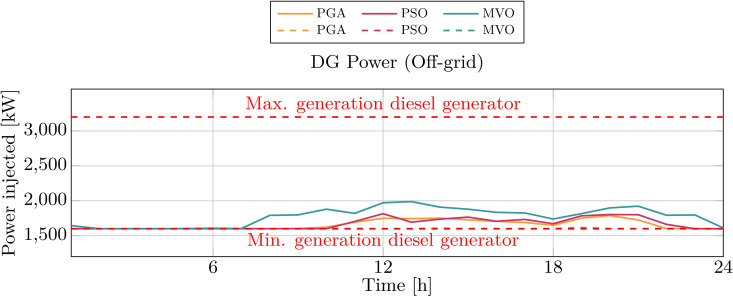
Performance metrics in the 33-node AC MG: Diesel generator power in off-grid mode (solid lines correspond to loss minimization, while dashed lines correspond to emission minimization).

To demonstrate that the applied metaheuristic techniques satisfy the technical constraints of the AC MG in both on-grid and off-grid operating modes, Fig 12 presents the voltage profiles, Fig 13 the line current flows, and Fig 14 the diesel generator dispatch at the slack bus for the off-grid mode. All results correspond to the best solution obtained by each technique. The optimal solutions produced by each methodology ensure that all nodal voltages remain within the acceptable range of [0.92, 1.08] p.u. throughout the day. For both operating modes, the largest voltage deviations, particularly under the emission-minimization objective, occur at 01:00, 6:00, and 24:00. Nevertheless, owing to the reactive power injection and/or absorption, these deviations remain small, not exceeding ±0.04 p.u. at any time of day.

Regarding the line loadabilities, they must not exceed the current limit specified for each line of the MG, which is set to 100% in the following figures. In both on-grid and off-grid operating modes, the lines most likely to reach their current limits are lines 11, 14, and 30, corresponding to the segments connecting nodes 11–12, 14–15, and 30–31, respectively. This shows that, although the techniques comply with the technical restrictions, they aim to maximize power flow from the WTs and BESS units, which are primarily constrained by the current-flow limits of the aforementioned lines.

On the other hand, in the specific case of the MG operating in off-grid mode, a diesel generator is connected at the slack bus instead of the utility grid, and it must operate at all times within [40, 80]% of its nominal capacity. Due to the high power production from the WTs and BESS units, the MG keeps this diesel generator operating near its minimum generation limit for most of the day. This effect is even more pronounced under the emission-minimization objective, in which case the generation curve and the lower operating limit appear nearly superimposed in the figure.

#### 4.1.2 Active and reactive power dispatch by PGA.

**Fig 15:**
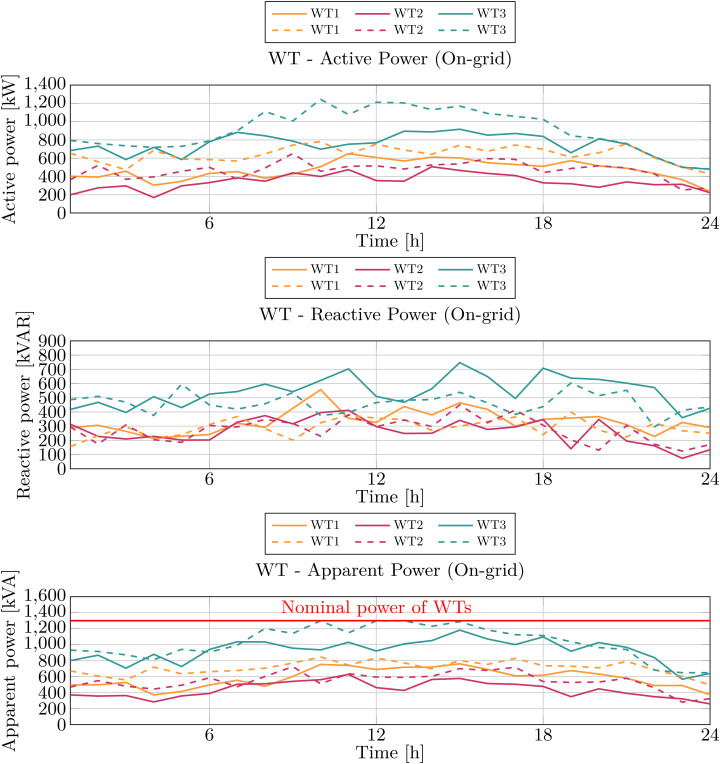
Power output from the WTs in the 33-node AC MG operating in on-grid mode (solid lines correspond to loss minimization, while dashed lines correspond to emission minimization).

**Fig 16:**
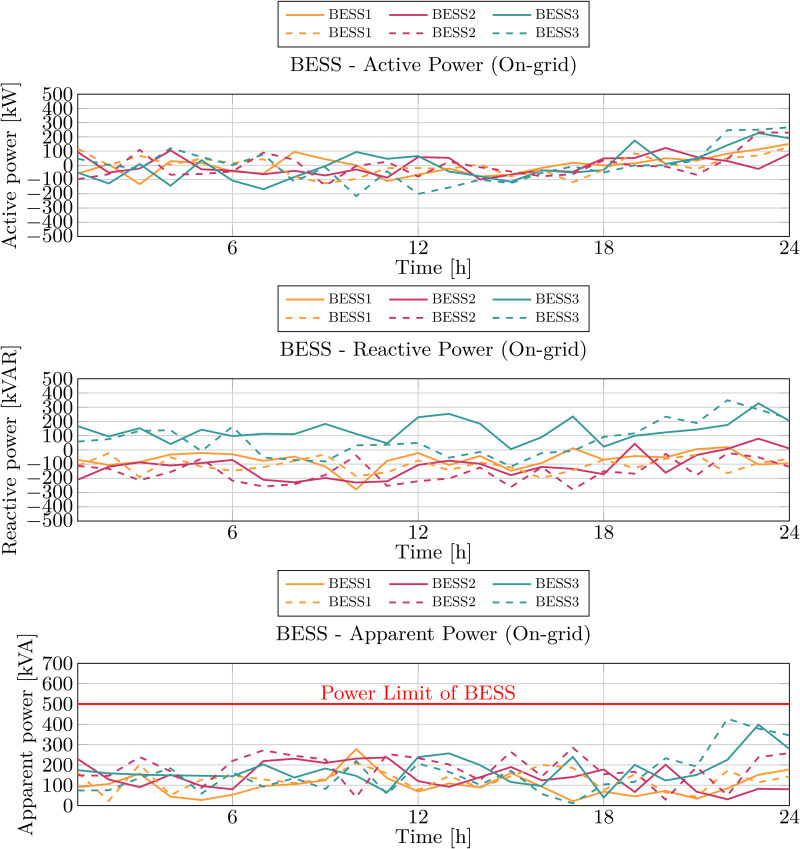
Power output from the BESS in the 33-node AC MG operating in on-grid mode (solid lines correspond to loss minimization, while dashed lines correspond to emission minimization).

**Fig 17:**
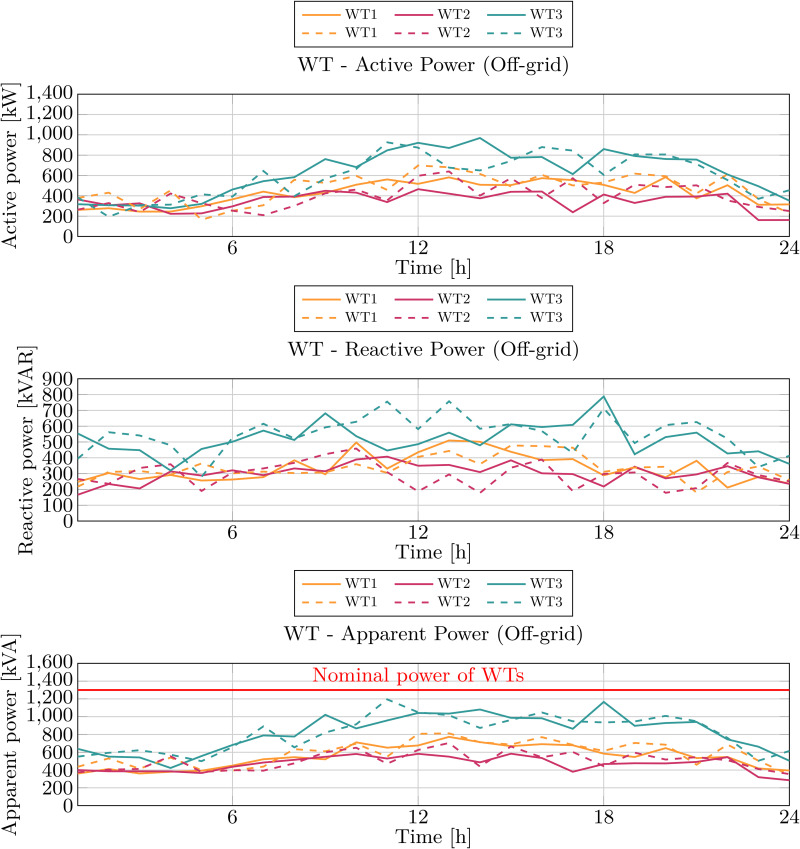
Power output from the WTs in the 33-node AC MG operating in off-grid mode (solid lines correspond to loss minimization, while dashed lines correspond to emission minimization).

**Fig 18:**
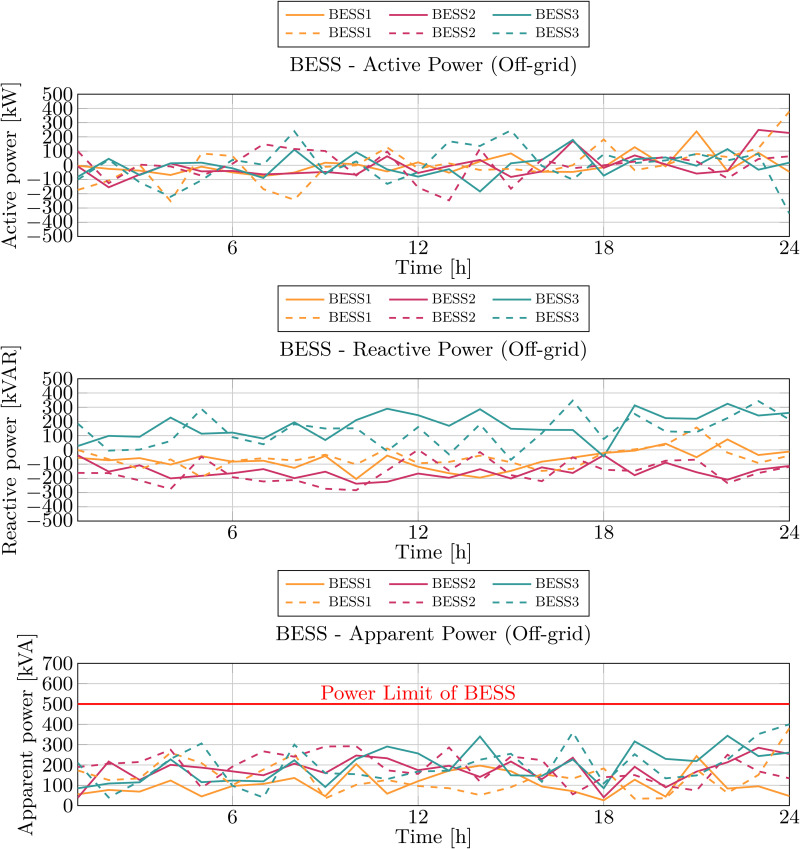
Power output from the BESSs (right) in the 33-node AC MG operating in off-grid mode (solid lines correspond to loss minimization, while dashed lines correspond to emission minimization).

In the remaining parts of the analysis for the 33-bus test system, only the results obtained with the best-performing methodology (PGA) are presented due to space limitations. The optimal scheduling reveals a coordinated interaction between wind generation and the BESS, aimed at maintaining feeder voltages close to their nominal values while reducing the current through heavily loaded branches. Since PGA achieved the best overall performance, the power dispatch plots are presented exclusively for this method. Figs 15 and 16 show the results obtained for the two objective functions considered in the on-grid case, whereas Figs 17 and 18 present the corresponding results for the off-grid mode.

In the loss-minimization case, the algorithm tends to dispatch additional reactive power from the BESS converter to mitigate line currents. Conversely, in the emission-minimization case, the BESS primarily exchanges active power to compensate for the temporal mismatch between wind production and demand. Moreover, in all scenarios, the WTs, particularly *WT*_3_ located at node 31, exhibit high power generation. In contrast, the BESS units inject relatively low power into the MG compared to their nominal ratings.

#### 4.1.3 Battery SOC dynamics obtained by PGA.

**Fig 19:**
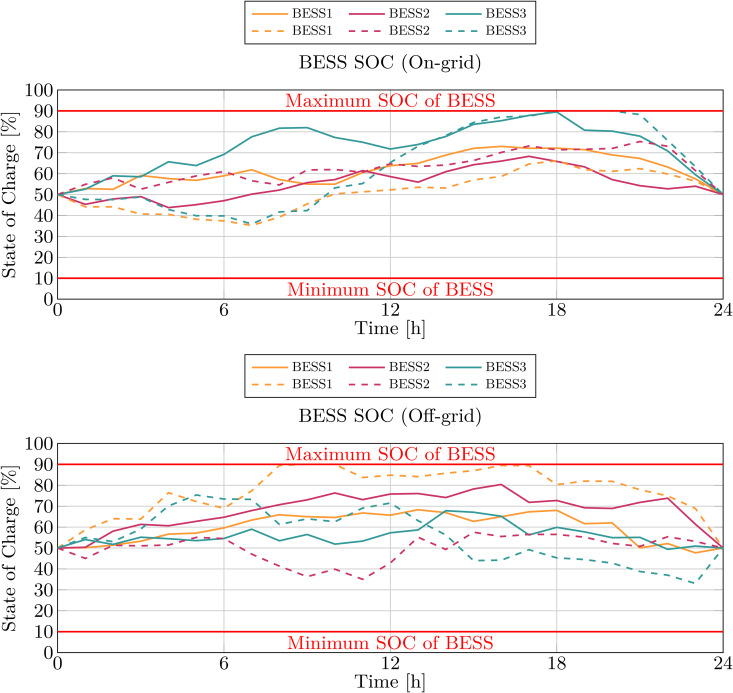
State of charge from the BESS in the 33-node AC MG operating in on-grid (left) and off-grid (right) modes (solid lines correspond to loss minimization, while dashed lines correspond to emission minimization).

The SOC trajectory follows a smooth daily pattern constrained between 10% and 90%, thus respecting the battery limitations in both the on-grid and off-grid operating modes of the MG, as shown in Fig 19.

In loss-minimization mode, SOC excursions remain more moderate because the device prioritizes reactive support over deep discharge cycles. Conversely, in the emission-minimization case, the BESS discharges more aggressively during high carbon–intensity hours, resulting in a steeper SOC decline. The SOC evolution further shows that the algorithm maintains sufficient charging reserves to ensure feasible operation throughout the entire horizon.

#### 4.1.4 Operation Analysis Including Battery Lifetime and Operational Efficiency.

The previous SOC model is used as the baseline formulation for the comparative benchmark on the 33-bus system. Subsequently, an extended BESS model that includes charging and discharging efficiencies, self-discharge, and lifetime estimation is introduced to assess the effects of these factors on the resulting schedules. In addition, the impact of battery operation on its lifetime was also analyzed. By integrating these efficiency-related factors, it was possible to identify their influence on both the objective functions and the battery lifetime during MG operation under on-grid and off-grid modes.

To account for the effects of charging and discharging efficiencies, the equation employed to update the battery state of charge, presented in Eq. (7), was modified by using Eq. (16). In this Equation, the SOC of battery *j* is modeled to account for the effects of power exchange, efficiency losses, and self-discharge.


$$\begin{array}{c} {SOC}_{j,t+1}={SOC}_{j,t}-\phi_{j}\;P_{B,j,t}^{aux}\;\Delta t-{SOC}_{j}^{AD}\;\Delta t, \end{array}$$ (16)

In the last equation, ${SOC}_{j,t}$ and ${SOC}_{j,t+1}$ denote the SOC at times *t* and *t* + 1, respectively, $\phi_{j}$ is the inverse of the nominal energy capacity of the ba*tt*ery, Δt is *t*he duration of the time step, and ${SOC}_{j}^{AD}$ represents the self-discharge rate. The term $P_{B,j,t}^{aux}$ corresponds to the battery power adjusted by efficiency, which depends on the operating mode of the device. It is defined as:


$$\begin{array}{c} P_{B,j,t}^{aux}=\left\{ \begin{array}{cc} P_{B,j,t}\;\eta_{c,j}\;\eta_{conv,j}, & P_{B,j,t}\leq 0,\\ \frac{P_{B,j,t}}{\eta_{d,j}\;\eta_{conv,j}}, & P_{B,j,t}>0, \end{array}\right. \end{array}$$ (17)

where $P_{B,j,t}$ is the scheduled battery power, $\eta_{c,j}$ is the charging efficiency, $\eta_{d,j}$ is the discharging efficiency, and $\eta_{conv,j}$ is the efficiency of the power converter.

This formulation captures the physical behavior of the battery during both charging and discharging processes. When $P_{B,j,t}\leq 0$, the battery operates in charging mode and only a fraction of the absorbed energy is effectively stored due to efficiency losses; therefore, the power is multiplied by the corresponding efficiencies. Conversely, when $P_{B,j,t}>0$, the battery operates in discharging mode and must internally supply more energy than the useful energy delivered to the system, which is represented by dividing the power by the discharging and conversion efficiencies.


$$\begin{array}{c} {\text{LifeB}}_{j}=\frac{2\;C_{b,j}\;N_{c,j}\;\lambda_{j}}{365\sum_{t=1}^{T}\left(\left|P_{j,t}^{\text{adj}}\right|+P_{j}^{AD}\right)\Delta h}. \end{array}$$ (18)

The lifetime estimation is carried out using an energy-throughput approach [34], in which the total usable energy each battery can deliver over its service life is compared with the annual energy processed under the studied operating profile. Accordingly, the lifetime of battery *j* in years is computed using Eq. (18). Where $C_{b,j}$ is the nominal energy capacity of battery *j*, $N_{c,j}$ is the number of life cycles assigned to that battery, $\lambda_{j}$ is the usable energy fraction of the battery capacity, $P_{j}^{AD}$ represents the constant power associated with the self-discharge of battery *j*, Δh is the time-step length in hours, *T* is the number of intervals in the daily horizon, and $P_{e,j}^{\text{adj}}$ is the adjusted battery power at time step *t*. In this work, $\lambda_{u}=0.8$, which represents the admissible usable fraction of the battery capacity. This factor is adopted to reflect that lithium-ion batteries are not operated over their full nominal storage range, but only within a prac*t*ical usable interval.

The nominal energy capacity is defined using Eq. (19), where $\phi_{j}$ is the inverse capacity parameter used in the implementation.


$$\begin{array}{c} C_{b,j}=\frac{1}{\phi_{j}}. \end{array}$$ (19)

The adjusted battery power is defined by implementing Eq. (20), where $P_{j,t}$ is the scheduled battery power and $\eta_{d}\eta_{conv,j}$ is the discharge efficiency. Positive values of $P_{j,t}$ represent discharge, while negative values correspond to charging. This correction is applied only during discharge because, due to efficiency losses, the battery must internally provide more energy than the useful energy effectively delivered to the system. Therefore, dividing the discharge power by $\eta_{d,j}\;\eta_{conv,j}$ allows the model to account for the actual energetic effort required from the battery.


$$\begin{array}{c} P_{e,t}^{\text{adj}}=\left\{ \begin{array}{cc} \frac{P_{j,t}}{\eta_{d,j}\;\eta_{conv,j}}, & P_{j,t}>0,\\ P_{j,t}, & P_{j,t}\leq 0. \end{array}\right. \end{array}$$ (20)

The lifetime expression accounts for the fact that a complete operating cycle includes both charging and discharging processes. Thus, the model estimates battery lifetime by comparing the total usable lifetime energy with the annual energy processed under the given daily power profile, considering that the analyzed operation represents an average day over a year and, therefore, its cumulative effect on battery degradation over time, ultimately expressing the lifetime in years. It should be noted that the BESS lifetime indicator adopted in this work is a first-order throughput-based approximation. Therefore, it does not represent a full electrochemical aging model. Temperature effects, C-rate dependency, calendar aging, internal resistance growth, and depth-of-discharge stress functions are not explicitly included. As a result, the obtained lifetime values should be interpreted as comparative indicators of battery utilization under the evaluated schedules, rather than as absolute lifetime predictions.

To analyze the performance of the proposed methodology over a representative time horizon, incorporating battery efficiency and lifetime considerations, the results are presented in Table 4. For this purpose, the average battery lifetime is also evaluated under a lossless scenario, in which both charging and discharging efficiencies are assumed to be 100%. For both analyses, a total of 3000 cycles over the battery lifetime was assumed, based on average technical data reported for commercial lithium-ion batteries [35].

**Table 4:** Comparison between the PGA results without and with efficiency modeling. Battery lifetime is reported in the following order: BESS1 (Bus 12), BESS2 (Bus 15), and BESS3 (Bus 31).

Category	Scenario	Base case	PGA without efficiencies	PGA with efficiencies	Battery lifetime (years)
Energy losses	On-grid	3378.92 kWh	491.34 kWh	494.3699 kWh	Without: [19.9471, 18.4624, 12.4192]
					With: [15.2121, 17.1104, 13.0205]
Energy losses	Off-grid	—	541.46 kWh	545.8976 kWh	Without: [20.0168, 15.5468, 11.6720]
					With: [10.0887, 13.1543, 16.5068]
CO_2_ emissions	On-grid	12541.2 kg CO_2_	4139.69 kg CO_2_	4196.8441 kg CO_2_	Without: [21.2842, 15.3955, 17.3586]
					With: [15.7606, 19.8847, 14.5092]
CO_2_ emissions	Off-grid	—	10257.77 kg CO_2_	10265.5207 kg CO_2_	Without: [11.6965, 13.5014, 10.5695]
					With: [16.5207, 14.8419, 11.7041]

**Fig 20:**
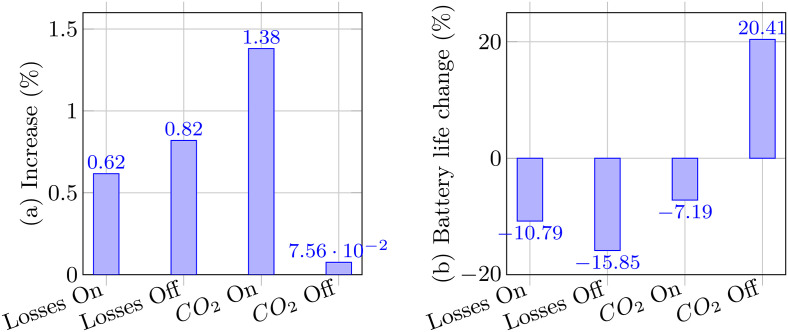
Impact of efficiency modeling on the optimized technical/environmental indicators and on the average battery lifetime: (a) Relative increase in the optimized objective value when efficiencies are considered and (b) Relative variation in the average battery lifetime after including efficiencies.

Fig 20 shows the impact of including battery and converter efficiencies in the optimization model. Regarding the main objective values, the inclusion of efficiencies resulted in small but systematic increases across all cases. For the energy-loss minimization problem, the optimized losses increased by 0.62% in the on-grid case and by 0.82% in the off-grid case. For the CO_2_ minimization problem, the optimized emissions increased by 1.38% in the on-grid case and by 0.08% in the off-grid case. On average, the increase was approximately 0.72% for energy losses and 0.73% for CO_2_ emissions.

Regarding battery degradation, the effect was more pronounced than that observed for the optimized objective values. The average battery lifetime decreased from 16.94 to 15.11 years in the on-grid loss-minimization case, from 15.75 to 13.25 years in the off-grid loss-minimization case, and from 18.01 to 16.72 years in the on-grid CO_2_-minimization case. These reductions correspond to relative changes of −10.79%, −15.85%, and −7.19%, respectively. In contrast, for the off-grid CO_2_-minimization case, the average battery lifetime increased from 11.92 to 14.36 years, corresponding to a relative improvement of 20.41%. This behavior can be explained by changes in optimal power dispatch when efficiency losses are explicitly considered. In this scenario, the inclusion of battery and converter efficiencies penalizes excessive charging and discharging actions, leading the optimization algorithm to reduce the overall energy throughput of the batteries. As a result, the batteries operate with smoother power profiles and lower cycling intensity.

Overall, when all cases are considered jointly, the mean battery lifetime decreased from 15.66 years to 14.86 years, equivalent to an average reduction of about 5.0%. This indicates that neglecting efficiencies produces only modest deviations in the optimized losses and emissions, but can lead to a more noticeable underestimation of battery degradation.

Wider SOC limits increase the usable energy capacity of the BESS and provide greater flexibility to shift renewable energy across the day. However, very wide SOC windows may increase battery stress if degradation-aware terms are not included. In contrast, narrow SOC limits reduce the ability of the BESS to absorb wind surplus or support the system during high-demand periods, which may increase losses, curtailment, or diesel/grid dependence. Similarly, larger BESS capacities generally improve energy-shifting capability, but may reduce marginal benefits once the main renewable surplus periods are already absorbed. Finally, the emission factor directly affects the CO_2_ oriented objective. Higher grid or diesel emission factors increase the incentive to use local renewable generation and stored energy, while lower emission factors reduce the relative environmental benefit of BESS dispatch.

#### 4.1.5 Operational analysis under an uncertainty scenario.

The performance of the PGA under higher variability and uncertainty conditions is evaluated using a one-week operational scenario based on the generation and demand profiles presented in Fig 4. In addition, the charging and discharging efficiencies of the batteries are considered to provide a more realistic representation of system operation. The corresponding results and analyses are presented in Tables 5 and 6.

**Table 5:** Weekly energy loss reduction for the 33-bus test system.

Operation Mode	Case	Monday	Tuesday	Wednesday	Thursday	Friday	Saturday	Sunday
Connected	Base Case	3071.4614	3071.4614	2927.1718	3071.4614	3071.4614	2906.1653	2734.6501
	PGA	454.8314	451.1854	431.5876	451.5667	454.8011	427.2489	402.0906
Isolated	PGA	459.7306	456.4826	436.0618	457.0358	459.7710	430.1377	403.6944

**Table 6:** Weekly CO_2_ emission reduction for the 33-bus test system.

Operation Mode	Case	Monday	Tuesday	Wednesday	Thursday	Friday	Saturday	Sunday
Connected	Base Case	11726.4923	11726.4923	11488.0190	11726.4923	11726.4923	11482.3859	11176.3366
	PGA	4516.4564	3391.2509	4305.8743	3507.6417	4539.8139	3223.7448	3228.8433
Isolated	PGA	8293.5229	7385.9741	7932.5841	7442.0859	8309.8050	7049.2180	6895.2239

Table 5 presents the results obtained by the PGA for loss reduction in the 33-bus MG under the variable generation and demand scenarios. For the on-grid case, the PGA achieves average daily losses of 439.0445 kWh, corresponding to an average loss reduction of 85.2626% with respect to the base case. In contrast, for the off-grid operation mode, no base case is available for comparison; therefore, the analysis cannot be performed in the same manner. Nevertheless, the obtained results show average daily losses of 443.2734 kWh.

Regarding the computational performance of the PGA, the average processing times were 162.4677 and 162.15785 seconds for the on-grid and off-grid operating modes, respectively.

An analysis of the average battery lifetime shows values of 20.1835, 22.4504, and 15.5663 years for the batteries installed at buses 12, 15, and 31, respectively, under the on-grid operating mode. In contrast, for the off-grid mode, the corresponding average lifetimes are 17.2136, 15.5685, and 14.1032 years for the same buses. These relatively high lifetime values suggest that, under the considered variable generation and demand scenarios, the BESSs may be oversized for the specific 33-bus MG under study, as their utilization is not sufficient to significantly accelerate battery degradation.

The results related to emission reduction are presented in Table 6. For the on-grid operating mode, average weekly emissions of 3816.2322 kgCO_2_ are obtained, corresponding to an average reduction of 67.0417% compared to the weekly base case. In contrast, for the off-grid mode, average weekly emissions of 7615.4877 kgCO_2_ are recorded. Regarding the computational performance of the PGA, the average processing time for the on-grid operating mode is 165.2123 seconds, while the off-grid operating mode requires an average processing time of 164.6607 seconds.

For the batteries installed at buses 12, 15, and 31, average lifetimes of 26.8628, 20.4859, and 20.5077 years are obtained under the on-grid operating mode. In the off-grid case, the corresponding average lifetimes are 15.1570, 16.5120, and 13.0136 years, respectively. These relatively high lifetime values, together with the even higher battery lifetimes obtained in the loss reduction study, suggest that, under the considered variable generation and demand scenarios, the BESSs may be oversized for the specific 33-bus MG under study, as their utilization is not sufficient to significantly accelerate battery degradation.

### 4.2 69-bus AC microgrid

To further evaluate the impact of the proposed methodology in a larger, more challenging scenario, this section analyzes the operation of the WT–BESS system in the 69-bus MG. Specifically, the study considers weekly demand and generation profiles, while accounting for uncertainty in both renewable power generation and load behavior under the on-grid and off-grid operating modes of the MG.

Only the PGA is employed in this stage, since the results obtained for the 33-bus system demonstrated that it is the best-performing methodology for solving the intelligent operation problem of WTs-BESS in AC MGs with the objective of reducing power losses and CO_2_ emissions. Additionally, this section analyzes the impact of battery efficiency on system performance and includes a battery lifetime assessment to provide a more comprehensive evaluation of the proposed operational strategy. The results obtained are presented in Tables 7 and 8.

**Table 7:** Weekly energy loss reduction for the 69-bus test system.

Operation Mode	Case	Monday	Tuesday	Wednesday	Thursday	Friday	Saturday	Sunday
Connected	Base Case	2030.6226	2030.6226	1936.4079	2030.6226	2030.6226	1922.8562	1810.5630
	PGA	219.8671	100.5228	198.6197	104.7234	221.7657	89.5689	105.8744
Isolated	PGA	238.5987	139.6924	200.4564	113.9037	228.3284	101.4284	111.1737

**Table 8:** Weekly CO_2_ emission reduction for the 69-bus test system.

Operation Mode	Case	Monday	Tuesday	Wednesday	Thursday	Friday	Saturday	Sunday
Connected	Base Case	9735.6324	9735.6324	9540.2165	9735.6324	9735.6324	9536.1623	9284.9067
	PGA	1894.5700	−1663.7605	1761.4749	−942.4340	1859.8314	−1898.7159	−565.4731
Isolated	PGA	5367.2764	4922.7662	5255.6427	5133.9668	5356.8696	4563.3907	5135.6940

First, Table 7 presents the results obtained by the PGA in reducing the energy losses of the 69-node MG. In on-grid mode, the optimization technique achieves average losses of 148.7060 kWh over a day of operation, which, compared to the average losses of the base case, corresponds to an approximate reduction of 92.4527%. For the off-grid mode, since there is no base case that excludes the implementation of the intelligent management of WTs and BESS, a comparison cannot be carried out in the same way; nevertheless, average losses of 161.9403 kWh are observed. Furthermore, an analysis of the average processing times yields values of 257.6157 and 300.1468 seconds for the on-grid and off-grid modes, respectively.

On the other hand, when analyzing the average battery lifetime under this strategy, values of 9.5021, 17.8205, and 7.0006 years are obtained for the batteries at nodes 15, 33, and 62, respectively, for the on-grid case, whereas for the off-grid case, these values are 8.6491, 11.0034, and 6.4700 years. This longer estimated lifetime may be associated with the reduced depth and frequency of charge and discharge cycles under the operating schedules obtained.

Some grid-connected scenarios in the 69-node MG present negative values of net CO_2_ emissions. These values should not be interpreted as negative physical emissions. They indicate that, during specific hours or days, the optimized WT-BESS schedule produces renewable surplus that is exported to the upstream grid. Under the net emission balance adopted in this study, exported renewable energy is accounted for as an emission credit. Therefore, negative values represent a net balance effect caused by grid export, while imported-grid emissions remain non-negative by definition.

Table 8 analyzes the impact of the proposed methodology on the weekly CO_2_ emissions reduction for the 69-bus test system. In the on-grid mode, an average emission level of 63.6418 kgCO_2_ is achieved, representing an average weekly reduction of 99.3381%. It is important to note that, on certain days, the resulting emissions take negative values. This can be interpreted as the MG, through intelligent resource management, generating more energy than it consumes and exporting the surplus to the main grid. In this sense, the strategy not only reduces the MG’s emissions but also lowers the emissions of the interconnected power system.

In off-grid mode, the MG achieves an average emission level of 5105.0867 kgCO_2_ while satisfying all technical constraints and operational limits of the system. Regarding computational performance, the average processing times are 262.5703 seconds for the on-grid case and 304.8200 seconds for the off-grid case. On the other hand, regarding battery lifetime, in the on-grid scenario, the batteries installed at nodes 15, 33, and 62 exhibit average lifetimes close to the maximum expected value of 25 years, respectively, due to their relatively low utilization. In contrast, in the off-grid operating mode, the corresponding lifetimes decrease to 9.2466, 8.8424, and 8.8588 years, respectively, due to more intensive battery use.

In all scenarios, the proposed methodology satisfies the operational limits imposed on the network and its components. Given the level of detail already provided for the 33-bus system, these results are omitted from this analysis.

## 5 Conclusions

This paper presented a day-ahead scheduling framework for the coordinated active and reactive power management of WTs and BESS units in AC MGs. The proposed formulation was applied to two independent objectives, namely energy-loss minimization and CO_2_ emission reduction, under both grid-connected and islanded operation. The framework was solved using PGA, PSO, and MVO under the same decision-variable encoding, feasibility correction, constraint handling, hourly AC power-flow evaluation, and 100-run statistical assessment.

The results obtained in the 33-node and 69-node test systems show that WT-BESS coordination can substantially improve the technical and environmental performance of AC MGs. The uncertainty analysis incorporated in both systems confirms that the proposed framework remains feasible under variable demand and wind-generation conditions. In addition, the statistical comparison shows that PGA provides a stable and repeatable performance in most evaluated cases, whereas PSO and MVO remain competitive depending on the objective function and operating mode.

The analysis also highlights several modeling considerations. First, negative values of net CO_2_ emissions in grid-connected scenarios correspond to export credits associated with renewable surplus, not to negative physical emissions. Second, the BESS lifetime indicator used in this study is a first-order throughput-based approximation and should be interpreted as a comparative utilization metric rather than as a complete electrochemical aging model. Third, the degradation model is not embedded in the optimization objective, which means that the proposed schedules should not be interpreted as degradation-aware optimal schedules.

Future work will address these limitations by incorporating degradation-aware objective terms based on cycle aging, throughput cost, C-rate effects, depth-of-discharge stress, and temperature-dependent degradation. In addition, future studies should explore Pareto-based formulations to reconcile loss and emission objectives in a single decision-making framework, deterministic optimization alternatives based on suitable AC power-flow approximations, and learning-based scheduling methods trained with historical renewable generation and demand data. Finally, the proposed framework should be validated using real Colombian distribution feeders and measured WT-BESS operating profiles.

## References

[1] Hoummadi MA, Aroussi HA, Bossoufi B, Karim M, Mobayen S, Zhilenkov A, et al. A review of constraints and adjustable parameters in microgrids for cost and carbon dioxide emission reduction. Heliyon. 2024;10(6):e27489. doi: 10.1016/j.heliyon.2024.e27489 38515729 PMC10955257

[2] Riou M, Dupriez-Robin F, Grondin D, Le Loup C, Benne M, Tran QT. Multi-Objective Optimization of Autonomous Microgrids with Reliability Consideration. Energies. 2021;14(15):4466. doi: 10.3390/en14154466

[3] Lan H, Wen S, Fu Q, Yu D, Zhang L. Modeling analysis and improvement of power loss in microgrid. Mathematical Problems in Engineering. 2015;2015(1):493560.

[4] Fresia M, Bordo L, Delfino F, Bracco S. Optimal day-ahead active and reactive power management for microgrids with high penetration of renewables. Energy Conversion and Management: X. 2024;23:100598. doi: 10.1016/j.ecmx.2024.100598

[5] Mohagheghi E, Alramlawi M, Gabash A, Blaabjerg F, Li P. Real-Time Active-Reactive Optimal Power Flow with Flexible Operation of Battery Storage Systems. Energies. 2020;13(7):1697. doi: 10.3390/en13071697

[6] Charalambous A, Hadjidemetriou L, Zacharia L, Bintoudi AD, Tsolakis AC, Tzovaras D, et al. Phase Balancing and Reactive Power Support Services for Microgrids. Applied Sciences. 2019;9(23). doi: 10.3390/app9235067

[7] Grisales-Noreña LF, Vega HP, Montoya OD, Botero-Gómez V, Sanin-Villa D. Cost Optimization of AC Microgrids in Grid-Connected and Isolated Modes Using a Population-Based Genetic Algorithm for Energy Management of Distributed Wind Turbines. Mathematics. 2025;13(5):704. doi: 10.3390/math13050704

[8] Llanos-Pino MA, Grisales-Noreña LF, Sanin-Villa D, Montoya OD, Hernández JC. Optimizing economic and operational performance in AC microgrids: An intelligent energy management strategy for BESS using the Generalized Normal Distribution Optimizer. Results in Engineering. 2025;:106005.

[9] Figueroa-Saavedra HA, Sanin-Villa D, Grisales-Noreña LF. A Tuned Parallel Population-Based Genetic Algorithm for BESS Operation in AC Microgrids: Minimizing Operational Costs, Power Losses, and Carbon Footprint in Grid-Connected and Islanded Topologies. Electricity. 2025;6(3):45. doi: 10.3390/electricity6030045

[10] Sanin-Villa D, Grisales-Noreña LF, Montoya OD. Coordinated Active–Reactive Power Scheduling of Battery Energy Storage in AC Microgrids for Reducing Energy Losses and Carbon Emissions. Sci. 2025;7(4):147. doi: 10.3390/sci7040147

[11] Grisales-Noreña L, Cortés-Caicedo B, Montoya OD, Sanin-Villa D, Gil-González W. Integration of BESS in grid connected networks for reducing the power losses and CO2 emissions: A parallel master-stage methodology based on PDVSA and PSO. Journal of Energy Storage. 2024;87:111355.

[12] Grisales-Noreña LF, Sanin-Villa D, Montoya OD. Optimal integration of PV generators and D-STATCOMs into the electrical distribution system to reduce the annual investment and operational cost: A multiverse optimization algorithm and matrix power flow approach. e-Prime - Advances in Electrical Engineering, Electronics and Energy. 2024;9:100747. doi: 10.1016/j.prime.2024.100747

[13] Hu Q, Zhao G, Hu J, Razmjooy N. Maximizing energy storage in Microgrids with an amended multi-verse optimizer. Heliyon. 2023;9(11):e21471. doi: 10.1016/j.heliyon.2023.e21471 37942149 PMC10628701

[14] Sanin-Villa D, Figueroa-Saavedra HA, Grisales-Noreña LF. Efficient BESS Scheduling in AC Microgrids via Multiverse Optimizer: A Grid-Dependent and Self-Powered Strategy to Minimize Power Losses and CO2 Footprint. ASI. 2025;8(3):85. doi: 10.3390/asi8030085

[15] Lobos-Cornejo S, Grisales-Noreña LF, Andrade F, Montoya OD, Sanin-Villa D. Smart Energy Strategy for AC Microgrids to Enhance Economic Performance in Grid-Connected and Standalone Operations: A Gray Wolf Optimizer Approach. Sci. 2025;7(2):73. doi: 10.3390/sci7020073

[16] Nagarajan K, Rajagopalan A, Angalaeswari S, Natrayan L, Mammo WD. Combined Economic Emission Dispatch of Microgrid with the Incorporation of Renewable Energy Sources Using Improved Mayfly Optimization Algorithm. Comput Intell Neurosci. 2022;2022:6461690. doi: 10.1155/2022/6461690 35479598 PMC9038389

[17] Tahmasebi M, Pasupuleti J, Mohamadian F, Shakeri M, Guerrero JM, Basir Khan MR, et al. Optimal Operation of Stand-Alone Microgrid Considering Emission Issues and Demand Response Program Using Whale Optimization Algorithm. Sustainability. 2021;13(14):7710. doi: 10.3390/su13147710

[18] Lamari M, Amrane Y, Boudour M, Boussahoua B. Multi‐objective economic/emission optimal energy management system for scheduling micro‐grid integrated virtual power plant. Energy Science & Engineering. 2022;10(8):3057–74. doi: 10.1002/ese3.1188

[19] Xing L, Liu Y. An optimization capacity design method of wind/photovoltaic/hydrogen storage power system based on PSO-NSGA-II. Energy Engineering: Journal of the Association of Energy Engineers. 2023;120(4):1023.

[20] Abdelwahab SAM, El-Rifaie AM, Hegazy HY, Tolba MA, Mohamed WI, Mohamed M. Optimal Control and Optimization of Grid-Connected PV and Wind Turbine Hybrid Systems Using Electric Eel Foraging Optimization Algorithms. Sensors (Basel). 2024;24(7):2354. doi: 10.3390/s24072354 38610565 PMC11014304

[21] Gamil MM, Masrur H, Muttaqi KM, Huang Y, Lotfy ME, Senjyu T. Multi-objective Optimal Power Scheduling of A Residential Microgrid Considering V2G and Demand Response Techniques. In: 2022 IEEE Industry Applications Society Annual Meeting (IAS), 2022. 1–5. 10.1109/ias54023.2022.9939886

[22] Werkie YG, Kefale HA. Optimal allocation of multiple distributed generation units in power distribution networks for voltage profile improvement and power losses minimization. Cogent Engineering. 2022;9(1). doi: 10.1080/23311916.2022.2091668

[23] Kalidasan M, Guna Sekar T, Mohanasundaram T. Power loss reduction and voltage profile enhancement by reconfiguration of radial distribution system with hybrid optimization method. Thermal Science and Engineering Progress. 2025;57:103105. doi: 10.1016/j.tsep.2024.103105

[24] Aščerić A, Čepin M. Improving power distribution system reliability via optimized microgrid integration and storage management. Reliability Engineering & System Safety. 2025;:111386.

[25] Honghai K, Fuqing S, Yurui C, Kai W, Zhiyi H. Reactive power optimization for distribution network system with wind power based on improved multi-objective particle swarm optimization algorithm. Electric Power Systems Research. 2022;213:108731. doi: 10.1016/j.epsr.2022.108731

[26] Igunma TO, Etukudoh EA. Innovations in battery energy storage systems (BESS) for sustainable renewable energy solutions. Innovations. 2025;9(4):430–8.

[27] Hosseini E, Horrillo-Quintero P, Carrasco-Gonzalez D, García-Triviño P, Sarrias-Mena R, García-Vázquez CA, et al. Optimal energy management system for grid-connected hybrid power plant and battery integrated into multilevel configuration. Energy. 2024;294:130765. doi: 10.1016/j.energy.2024.130765

[28] Tanha MH, Tanha Z, Aranizadeh A, Mirmozaffari M. Sustainable Wind Energy Security: Assessing the Impact of False Data Injection on Wind Turbine Performance. Sustainability. 2025;17(10):4654. doi: 10.3390/su17104654

[29] Aranizadeh A, Vahidi B, Rahiminejad A. Wind turbine power output smoothing in microgrid using ultra-capacitor with continuous wind speed forecasting and online supervisory control. Journal of Renewable and Sustainable Energy. 2016;8(3). doi: 10.1063/1.4950958

[30] Aranizadeh A, Zaboli A, Asgari Gashteroodkhani O, Vahidi B. Wind turbine and ultra-capacitor harvested energy increasing in microgrid using wind speed forecasting. Engineering Science and Technology, an International Journal. 2019;22(5):1161–7. doi: 10.1016/j.jestch.2019.08.006

[31] Issa M, Ibrahim H, Hosni H, Ilinca A, Rezkallah M. Effects of Low Charge and Environmental Conditions on Diesel Generators Operation. Eng. 2020;1(2):137–52. doi: 10.3390/eng1020009

[32] Cubillo-Leyton PI, Montoya OD, Grisales-Noreña LF. Optimized integration of photovoltaic systems and distribution static compensators in distribution networks using a novel discrete-continuous version of the adaptive JAYA algorithm (AJAYA). Results in Engineering. 2025;26:104726. doi: 10.1016/j.rineng.2025.104726

[33] Bolaños RI, Pinto Vega H, Grisales-Noreña LF, Montoya OD, Hernández JC. Intelligent Active and Reactive Power Management for Wind-Based Distributed Generation in Microgrids via Advanced Metaheuristic Optimization. ASI. 2025;8(4):87. doi: 10.3390/asi8040087

[34] Khojasteh M, Faria P, Vale Z. Energy-constrained model for scheduling of battery storage systems in joint energy and ancillary service markets based on the energy throughput concept. International Journal of Electrical Power & Energy Systems. 2021;133:107213. doi: 10.1016/j.ijepes.2021.107213

[35] Victron Energy B V. Lithium-ion Battery and Lynx Ion Shunt Datasheet. 2024. https://www.victronenergy.com.es/upload/documents/Datasheet-Lithium-ion-and-Lynx-Ion-ES.pdf

